# Extended Combined Neonatal Treatment With Erythropoietin Plus Melatonin Prevents Posthemorrhagic Hydrocephalus of Prematurity in Rats

**DOI:** 10.3389/fncel.2018.00322

**Published:** 2018-09-25

**Authors:** Shenandoah Robinson, Fatu S. Conteh, Akosua Y. Oppong, Tracylyn R. Yellowhair, Jessie C. Newville, Nagat El Demerdash, Christine L. Shrock, Jessie R. Maxwell, Stephen Jett, Frances J. Northington, Lauren L. Jantzie

**Affiliations:** ^1^Division of Pediatric Neurosurgery, School of Medicine, Johns Hopkins University, Baltimore, MD, United States; ^2^Department of Pediatrics, University of New Mexico Health Sciences Center, Albuquerque, NM, United States; ^3^Department of Neurosciences, University of New Mexico Health Sciences Center, Albuquerque, NM, United States; ^4^Department of Cell Biology and Physiology, University of New Mexico Health Sciences Center, Albuquerque, NM, United States; ^5^Division of Neonatology, School of Medicine, Johns Hopkins University, Baltimore, MD, United States

**Keywords:** intraventricular hemorrhage (IVH), chorioamnionitis, neurorepair, ventriculomegaly, diffusion tensor imaging (DTI), preterm, cilia, cerebrospinal fluid

## Abstract

Posthemorrhagic hydrocephalus of prematurity (PHHP) remains a global challenge. Early preterm infants (<32 weeks gestation), particularly those exposed to chorioamnionitis (CAM), are prone to intraventricular hemorrhage (IVH) and PHHP. We established an age-appropriate, preclinical model of PHHP with progressive macrocephaly and ventriculomegaly to test whether non-surgical neonatal treatment could modulate PHHP. We combined prenatal CAM and postnatal day 1 (P1, equivalent to 30 weeks human gestation) IVH in rats, and administered systemic erythropoietin (EPO) plus melatonin (MLT), or vehicle, from P2 to P10. CAM-IVH rats developed progressive macrocephaly through P21. Macrocephaly was accompanied by ventriculomegaly at P5 (histology), and P21 (*ex vivo* MRI). CAM-IVH rats showed impaired performance of cliff aversion, a neonatal neurodevelopmental test. Neonatal EPO+MLT treatment prevented macrocephaly and cliff aversion impairment, and significantly reduced ventriculomegaly. EPO+MLT treatment prevented matted or missing ependymal motile cilia observed in vehicle-treated CAM-IVH rats. EPO+MLT treatment also normalized ependymal yes-associated protein (YAP) mRNA levels, and reduced ependymal GFAP-immunolabeling. Vehicle-treated CAM-IVH rats exhibited loss of microstructural integrity on diffusion tensor imaging, which was normalized in EPO+MLT-treated CAM-IVH rats. In summary, combined prenatal systemic inflammation plus early postnatal IVH caused progressive macrocephaly, ventriculomegaly and delayed development of cliff aversion reminiscent of PHHP. Neonatal systemic EPO+MLT treatment prevented multiple hallmarks of PHHP, consistent with a clinically viable, non-surgical treatment strategy.

## Introduction

Posthemorrhagic hydrocephalus of prematurity (PHHP) is a severe form of acquired symptomatic hydrocephalus. PHHP, the combination of ventriculomegaly and elevated intracranial pressure, occurs most commonly in preterm infants who suffer severe intraventricular hemorrhage (IVH) during the first few days of life. Severe IVH is defined as germinal matrix hemorrhage distending a ventricle or involving adjacent parenchyma. Worldwide, ∼1.6 million early preterm infants (<32 weeks gestation) are born annually (Blencowe et al., 2012). In countries with advanced medical care, 8–23% of these early preterm infants (∼300,000 infants per year) are afflicted by severe IVH (Alan et al., 2012; Radic et al., 2015; Romero et al., 2015; Poryo et al., 2017). Symptomatic PHHP typically develops weeks after IVH, and represents the worst outcome along a spectrum of severity after severe IVH. The spectrum ranges from ventriculomegaly without signs of elevated intracranial pressure to symptomatic PHHP that requires an invasive intervention. Of surviving early preterm infants with severe IVH, 50–60% will exhibit ventriculomegaly on head ultrasound (HUS) during the newborn admission (Alan et al., 2012; Romero et al., 2015), 20–30% will develop symptomatic hydrocephalus that requires temporary treatment (Alan et al., 2012; Radic et al., 2015), and 16–20% will require permanent intervention (Alan et al., 2012; Radic et al., 2015; Romero et al., 2015). Currently, the only effective treatment for PHHP is surgical intervention (Mazzola et al., 2014), yet most infants and children throughout the world do not have access to safe, timely neurosurgical care (Muir et al., 2016). In the United States, pediatric hydrocephalus treatment consumes $1.4–2 billion dollars annually (2003 dollars) (Simon et al., 2008). The most common neurosurgical intervention for PHHP, insertion of a permanent cerebrospinal fluid (CSF) shunt, brings lifelong dependence on devices prone to malfunction and infection. In a recent multi-center study in the United States, PHHP was the most common reason for initial shunt insertion, and one third of initial infant shunts failed with a mean time to failure of <1 year (Riva-Cambrin et al., 2016). Shunt outcomes are much worse in regions without advanced neurosurgical care (Santos et al., 2017). Other surgical options for infants, such as endoscopic third ventriculostomy with choroid plexus coagulation (ETV-CPC), have not shown superior durability to shunts (Kulkarni et al., 2018). The need for safe, effective, economical, non-surgical treatment of PHHP cannot be over-stated.

Over the past two decades, perinatal neuroreparative strategies have been translated into clinical trials (Johnston et al., 2011; Leuchter et al., 2014; Ohls et al., 2014; Wu et al., 2016; Juul et al., 2018) and practice (Gluckman et al., 2005). This progress motivated us to re-examine non-surgical treatment of PHHP. The pluripotent cytokines, erythropoietin (EPO) and melatonin (MLT), are leading candidates for neurorepair in neonates with central nervous system (CNS) injury due to their safety profile (Robertson et al., 2012), and multiple and complementary, mechanisms of action on developing neural cells (Brines et al., 2000; Carloni et al., 2008; Mazur et al., 2010; Jantzie et al., 2018). Both EPO and MLT support the genesis, survival and maturation of neural cells (Iwai et al., 2010; Mazur et al., 2010; Jantzie et al., 2013; Li et al., 2017; Zhang et al., 2018), reduce excess calpain degradation (Samantaray et al., 2008; Jantzie et al., 2014b, 2016; Robinson et al., 2016), limit neuroinflammation and oxidative damage (Carloni et al., 2016; Ramirez-Jirano et al., 2016; Dominguez Rubio et al., 2017; McDougall et al., 2017; Wang et al., 2017; Wei et al., 2017; Zhou et al., 2017), and suppress endoplasmic reticulum stress and mitochondrial dysfunction (Hong et al., 2012; Das et al., 2013; Carloni et al., 2014; Fernandez et al., 2015; Hadj Ayed Tka et al., 2015; Zhao et al., 2015; Hu et al., 2016; Hardeland, 2017; Mendivil-Perez et al., 2017; Tang et al., 2017; Xue et al., 2017). Individually, EPO and MLT are in clinical trials to ameliorate CNS injury from extremely preterm birth, demonstrating their clinical viability as therapeutics in preterm infants (Juul et al., 2008; Ohls et al., 2014, 2016; Fauchere et al., 2015; Wu et al., 2016; Carloni et al., 2017). A multi-pronged therapeutic approach is commonly used for other medical disorders that have multiple interacting targets, and may benefit PHHP (Gonzalez and Ferriero, 2008).

The association of intrauterine inflammation, or chorioamnionitis (CAM), with IVH has been recognized around the world (Moscuzza et al., 2011; Salas et al., 2013; Arayici et al., 2014; Shankaran et al., 2014; Lee et al., 2016; Lu et al., 2016; Stark et al., 2016; Chevallier et al., 2017; Edwards et al., 2018). In a recent study, 26.7% of early preterm infants with CAM exposure suffered IVH, compared to only 17.8% of infants without CAM exposure (Edwards et al., 2018). Systemic inflammation is also implicated in the need for surgical intervention for PHHP (Alan et al., 2012). In a longitudinal study of early preterm infants, the incidence of IVH remained stable at 8% for over a decade (Alan et al., 2012). By contrast, the incidence of sepsis dropped significantly. Interestingly, the need for surgical intervention for PHHP declined and followed the sepsis trend, rather than remaining stable and trending with IVH (Alan et al., 2012). Decades ago, Rypens et al. (1994) described widespread ependymal changes after IVH visualized throughout the ventricular system on HUS. These changes were not restricted to areas of IVH, implicating a diffuse ependymal reaction after IVH. Together, these clinical studies suggest associations between prenatal systemic inflammation that impacts the developing CNS, an increased risk of IVH, diffuse ependymal reaction following IVH, and the greater need for surgical intervention to treat symptomatic hydrocephalus.

Recent advances in neuroscience have shed new insights on the pathogenesis of symptomatic hydrocephalus. Ependymal motile cilia (EMC) propel CSF through the ventricles (Banizs et al., 2005; Sawamoto et al., 2006; Faubel et al., 2016). Ependymal cells arise from radial glia in the third trimester (Spassky et al., 2005), which in rodents is equivalent to approximately the first postnatal week (Semple et al., 2013; Jantzie and Robinson, 2015). The genesis, maturation and anatomical arrangement of EMC is complex and strictly regulated during development, primarily from postnatal day 3 (P3) through 21 in rats (Banizs et al., 2005; Guirao et al., 2010; Mirzadeh et al., 2010; Tissir et al., 2010; Lattke et al., 2012; Delgehyr et al., 2015; Kyrousi et al., 2015; Feldner et al., 2017; Takagishi et al., 2017; Abdelhamed et al., 2018; Cho et al., 2018; Mahuzier et al., 2018). Development of EMC occurs precisely during the perinatal period when early preterm infants suffer associated CNS injury from CAM and IVH (Delgehyr et al., 2015). After IVH in human preterm infants, ependymal cells lining the ventricles are replaced by astrocytic glial nodules in a process termed ventricular disruption (Fukumizu et al., 1995, 1996; McAllister et al., 2017). Similarly, EMC injury occurs following intracerebral hematoma (Chen et al., 2015), traumatic brain injury (TBI) (Xiong et al., 2014) and stroke (Young et al., 2013). Converging lines of evidence suggest that damage to EMC leads to excessive accumulation of CSF and symptoms of elevated intracranial pressure.

We propose EMC failure leading to symptomatic hydrocephalus is precipitated by systemic inflammation in combination with a second CNS insult, such as hemorrhage or TBI. Yung et al. (2011) demonstrated that fetal intraventricular lysophosphatidic acid (LPA), a blood-borne inflammatory signaling molecule, precipitates congenital hydrocephalus, and Park subsequently showed that LPA reduces yes-associated protein (YAP), and ependymal maturation (Park et al., 2016). YAP drives the differentiation of radial glial cells toward ependymal cells (Park et al., 2016). Without adequate YAP present, radial glial cells become astrocytes (Huang et al., 2016). We hypothesize that CAM plus early IVH induces symptomatic hydrocephalus that replicates human infant PHHP, and that this occurs in part through alteration of periventricular YAP. Moreover, we propose that combined treatment with EPO and MLT can prevent EMC damage, and thus PHHP. Herein, we report a novel, clinically relevant model of PHHP that combines prenatal injury to induce CNS inflammation plus early postnatal IVH, and prevention of PHHP with neonatal EPO+MLT treatment. This clinically viable, non-surgical intervention prevents acquired hydrocephalus in a preclinical model that exhibits macrocephaly, ventriculomegaly and developmental delay (poor cliff aversion performance), mimicking symptomatic PHHP in human infants.

## Materials and Methods

### Study Design

The primary study objective was to determine if progressive macrocephaly and ventriculomegaly could be reliably induced in neonatal rats using a combination of CAM and IVH, and if combinatorial therapy with EPO+MLT could prevent these two hallmarks of PHHP. For each experiment with a statistical analysis, a power analysis was performed to estimate the required sample size (G^∗^Power 3.1.9.3). We used preliminary and published data to estimate means and standard deviations for each group for the primary outcomes of intra-aural distance, ventriculomegaly, neurodevelopmental assessment, qPCR, and DTI analyses (Farkas et al., 2009; Yung et al., 2011; Baharnoori et al., 2012; Jantzie et al., 2014a,b, 2018; Robinson et al., 2016). Scanning electron microscopy qualitative analysis of EMC was set *a priori* as two per group. Primary endpoints were set *a priori* as described in the text, and were daily (P4-P15 and P21) for IAD and neurodevelopment, P5 for ventriculomegaly, P15 for periventricular YAP mRNA, and P21 for remaining assays. No animals were individually excluded except, as noted in the text, those who were removed prior to P21 due to poor health. DTI images from one rat were excluded due to poor image acquisition quality prior to quantification. Rats from both institutions and at least two separate litters were used for all experiments, except rats for SEM were prepared at one institution. Rats from both sexes were randomly assigned to injury (CAM versus sham at E18, IVH versus vehicle at P1) and to treatment group (EPO+MLT or vehicle on P2 for CAM-IVH rats). Each rat pup was coded to ensure all observations and assays and data analyses were performed blinded prior to decoding for interpretation.

### Animals

**FIGURE 1:**
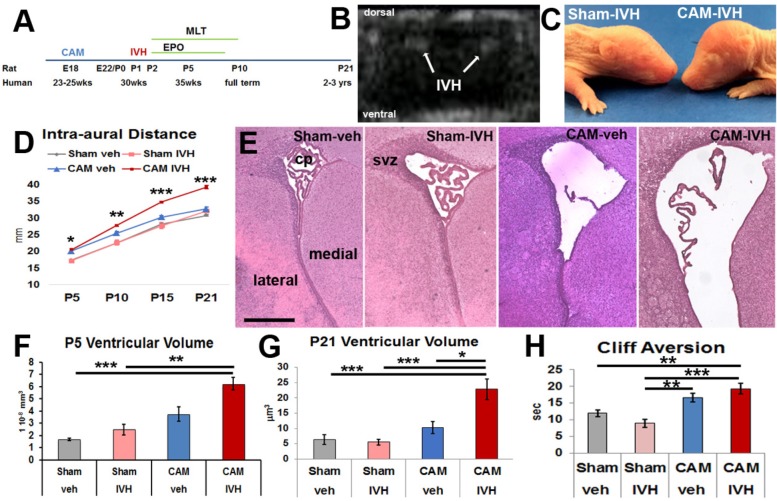
Prenatal chorioamnionitis (CAM) plus postnatal day 1 (P1) intraventricular injection of lysed red blood cells (IVH), or CAM-IVH, causes progressive macrocephaly, ventriculomegaly and neurodevelopmental delay. **(A)** Experimental paradigm. **(B)** Coronal head ultrasound on P2 demonstrating bilateral IVH. **(C)** A representative P7 rat with CAM-IVH exhibits a domed cranial vault, while a sham-IVH rat does not. **(D)** CAM-IVH rats show a disproportionate increase in intra-aural distance (IAD), a surrogate for head circumference, while control rats (sham-veh, sham-IVH and CAM-veh) do not. **(E)** Hematoxylin and eosin staining of coronal sections of the lateral ventricle at P5 demonstrate ventriculomegaly in CAM-IVH rats. Bar = 500 microns. **(F)** Ventricular volume measured with unbiased stereology of HE sections at P5 is mildly increased in CAM-veh rats, and markedly elevated in CAM-IVH rats, compared to sham-veh or sham-IVH rats. **(G)** Ventricular volume quantified from coronal *ex vivo* T2 MRI slices shows that ventriculomegaly in CAM-IVH rats is sustained through P21, compared to sham-veh, sham-IVH and CAM-veh rats. **(H)** Both CAM-veh and CAM-IVH rats exhibit delayed performance on cliff aversion, a neonatal neurodevelopmental test. (**D** is mixed model repeated measures ANOVA with Bonferroni’s correction and all other comparisons are two-way ANOVA with Bonferroni’s correction, ^∗^*p* < 0.05, ^∗∗^*p* ≤ 0.01, ^∗∗∗^*p* ≤ 0.001.)

All procedures followed the National Research Council guidelines, and were performed with the approval of IACUCs at Johns Hopkins University (RA16M369) and the University of New Mexico Health Sciences Center (16-200398-HSC). Timed pregnant Sprague-Dawley dams were purchased (Charles River). Rats had *ad libitum* food and water, and a standard 12-h light dark cycle. Pups were raised with dams and weaned at P21. Pups were monitored daily for weight and other signs of health. A total of 298 pups were used for this study. Both sexes, and rats from at least two separate litters were used for all experiments. The experimental paradigm is presented in **Figure 1A**. ARRIVE guidelines were followed (Kilkenny et al., 2010). An initial cohort of 135 rat pups [43 sham-vehicle (sterile saline), 35 sham-IVH, 27 CAM-vehicle, and 30 CAM-IVH] was used to establish the model of PHHP. A second cohort of 89 rats (31 sham-veh rats, 35 CAM-IVH vehicle-treated rats, and 23 CAM-IVH EPO+MLT-treated rats) was used to test if treatment begun on P2 could prevent the development of PHHP. A third cohort of 74 pups (10 naïve vehicle-treated rats, 20 naïve EPO+MLT-treated rats, 13 vehicle-treated CAM-vehicle rats, 8 vehicle-treated CAM-IVH rats, 5 MLT-treated CAM-IVH rats, 9 EPO-treated CAM-IVH rats, and 9 EPO+MLT-treated CAM-IVH rats) was used for additional control experiments. Whenever possible, each pup was used for multiple assays.

### Chorioamnionitis (CAM) on Embryonic Day 18

To induce CAM, under isoflurane anesthesia pregnant dams underwent laparotomy on embryonic day 18 (E18) with transient uterine artery occlusion for 60 min followed by intra-amniotic injection of lipopolysaccharide (LPS, 0111:B4, Sigma, St. Louis, MO, United States, 4 μl/sac) (Jantzie et al., 2014a, 2015b; Maxwell et al., 2015). The laparotomy was closed and pups were born on E22. Shams underwent anesthesia and laparotomy for 60 min without uterine artery occlusion or LPS injection. 44 dams underwent laparotomy to generate the animals for this study.

### Intraventricular Injection on Postnatal Day 1

For litters with IVH injection, one pup from each litter was randomly chosen as a donor for syngeneic lysed red blood cells (RBCs). Lysed RBCs were chosen because of prior work that suggested lysed RBCs best replicate human IVH (Strahle et al., 2012; Gao et al., 2014). Donor blood was collected, centrifuged at 500 ×*g* for 10 min, washed three times with sterile PBS (phosphate-buffered saline), and then lysed with a standardized series of freeze-thaw cycles. On postnatal day 1 (P1), CAM or sham pups were randomly assigned to injection of lysed RBCs (IVH) or vehicle (sterile saline, pH 7.4). The mean hematocrit of P1 lysed RBCs was 37.2 ± 0.9% for shams (*n* = 3) and 36.9 ± 0.6% for CAM rats (*n* = 3). Using hypothermia for sedation, transillumination, and standard landmarks for the lateral ventricle (0.2 mm anterior to the coronal suture and 1 mm lateral of midline), either vehicle or lysed RBCs (20 μl) was injected into each lateral ventricle using a micro-syringe with a 31 gauge needle (Kim et al., 2014; Mukai et al., 2017). Lysed RBCs or vehicle was slowly infused freehand with a percutaneous needle, as previously reported, because neonatal rats at P1 cannot be placed in a stereotactic frame (Mukai et al., 2017). Placement was confirmed visually with transillumination. After 15–20 s the needle was removed, and pups were rewarmed and returned to their dams. Rats were coded and labeled to blind observers for subsequent assessments. Ultrasound (VScan Extend Dual Probe, GE Healthcare) was used to confirm IVH on P2.

### Extended Erythropoietin (EPO) Plus Melatonin (MLT) Treatment

To test if EPO+MLT could prevent the onset of PHHP, on P2 CAM+IVH rats were randomized to receive either EPO+MLT or vehicle from P2 (equivalent to approximately 32 weeks human gestation) to P10 (full term in humans). Six doses of EPO (2500 U/kg/dose i.p.) or vehicle were administered on P2-P5, P7 and P9. Nine doses of MLT (20 mg/kg/dose i.p.) or vehicle were administered on P2-P10. A subset of 36 CAM-IVH pups were randomized to receive vehicle, EPO alone, MLT alone, or EPO+MLT to test impact of treatment on macrocephaly. Shams did not receive EPO+MLT because both EPO and MLT have previously been shown to be safe in these dosing regimens as individual agents (Juul et al., 2008; Mazur et al., 2010; Ohls et al., 2014; Fauchere et al., 2015; Carloni et al., 2017). To determine if EPO+MLT had any unanticipated effect on normal neurodevelopment, a set of naïve pups underwent EPO+MLT treatment. Doses were administered the same time of day to minimize any impact of MLT on the circadian rhythm, although no impact was evident.

### Intra-Aural Distance (IAD)

Observers blinded to injury and treatment group measured 200 rats (cohort 1 = 87, cohort 2 = 69, and cohort 3 = 44). Daily measurements included IAD (Yung et al., 2011) and body weight for dosing calculations.

### Neurodevelopmental Assessment

Neurodevelopmental battery included nine tests (surface righting, negative geotaxis, cliff aversion, forelimb grasp, audio startle, eye opening, ear twitch, forelimb placement, and air righting) (Farkas et al., 2009; Baharnoori et al., 2012). Since no differences with audio startle or eye opening were noted across groups, these two tests were not included for naïve rats. Observers blinded to injury and treatment group tested 146 rats each day from P4 to P15, and then at P21.

### Histology

Animals were anesthetized and perfused with PBS and then 4% paraformaldehyde (PFA), and brains were collected. After immersion in 30% sucrose, coronal frozen sections were cut on a cryostat. Hematoxylin and eosin (HE) staining, immunohistochemistry (IHC) and unbiased stereology were performed, as previously published (Jantzie et al., 2014a). Sections stained with HE were used for ventricular volume. For IHC, briefly, sections were washed and then incubated sequentially with 0.3% hydrogen peroxide and blocking solution containing 10% goat serum in PBS. Primary GFAP antibodies (Dako 1:500, Carpinteria, CA, United States) in blocking solution containing 0.1% Triton were incubated on sections overnight at 4°C. The next morning, sections were rinsed, incubated with species-appropriate biotinylated secondary antibodies, followed by Vectastain (Vector Labs, Burlingame, CA, United States) and 3′3′-diaminobenzidine (DAB). Sections were dehydrated, cleared in xylenes and cover-slipped with Permount (Millipore Sigma, St. Louis, MO, United States). Although dehydration can affect ventricular volume measurements in slide mounted sections, all sections were dehydrated equally, including equivalent times in graded ethanol solutions and xylenes. Appropriate negative controls (no primary antibodies) were run in parallel. Using bright-field illumination, representative images were photographed on a Leica microscope. Observers blinded to injury and treatment status quantified ventricular volumes with Stereologer software (Stereology Resource Center, Tampa, FL, United States) on an upright Leica microscope using optical fractionator methodology under the 40× objective.

### Quantitative PCR

Quantitative PCR was performed as previously published (Jantzie et al., 2014a). Primers for YAP were obtained from the literature (Huang et al., 2016), and verified with BLAST (National Center for Biotechnology Information Nucleotide Basic Local Alignment Search Tool). Briefly, ependyma at P15 were rapidly dissected for RNA isolation (RNeasy Mini kit; Qiagen, Germantown). Following extraction, RNA was transcribed to cDNA, followed by RT-PCR using SYBR green and YAP primers. Experimental replicates were run in triplicate and those CT values varying by more than 0.25 standard deviations were excluded from all analyses. Pooled adult naïve cortex was used to standardize across plates.

### MRI for Ventricular Volume and Microstructural Integrity

*Ex vivo* MRI T2 and diffusion tensor imaging (DTI) images were acquired at P21 on 33 rats using a Bruker Biospec 7T 70/30 Ultra Shield Refrigerated (USR) nuclear MRI system. Briefly, rats were anesthetized and perfused with PBS, followed by 4% PFA. Brains were post-fixed in 4% PFA for 1 week and embedded in 2% agarose containing 3 mM sodium azide. A T2 multi-slice multi-echo (MSME) sequence was performed with a TR of 3000 ms and TE of 12 ms. FOV was 3 cm × 3 cm, with a slice thickness of 1 mm, 12 slices total, and matrix of 256 × 256, as previously published (Jantzie et al., 2015a; Robinson et al., 2016). Echo-planar diffusion tensor imaging (EP-DTI) images of twelve contiguous coronal 1 mm slices was obtained with a FOV of 3. Ventricular volume was calculated from manually traced regions of interest (ROI) by observers blinded to injury and treatment status. Areas within each slice were calculated, summed across slices and multiplied by slice thickness. DTI was quantified for ROI consistent with previous publications (Jantzie et al., 2015a; Robinson et al., 2016). Because the intraventricular injections were performed bilaterally, for bilateral structures the metrics were acquired on each side and averaged per ROI for each rat.

### Scanning Electron Microscopy (SEM)

Six rats on P21 were perfused with PBS, followed by a mixture of 2.5% glutaraldehyde/2% PFA/0.1 M sodium cacodylate/HCl buffer pH 7.2. Anterior and posterior areas of the lateral or medial walls of the lateral ventricles were micro-dissected and fixed overnight. Anatomically matched tissues were incubated in 1% osmium tetroxide in 0.1 sodium cacodylate/HCl buffer for 4 h, dehydrated in a series of graded ethanol solutions, carbon-coated, then imaged in a Zeiss Sigma 300 field emission scanning electron microscope, consistent with published reports (Sawamoto et al., 2006; Xiong et al., 2014).

### Quantification and Statistical Analysis

In all studies, each rat represents true n. Observers were blinded to injury and treatment status for all data acquisition and initial analyses. For all data sets normal distribution was verified with the Shapiro–Wilk test, with Levene’s test to confirm homogeneity of variances. For comparisons of multiple groups that were normal in distribution, two-way ANOVA with Bonferroni’s correction was performed (SPSS 24, IBM). For comparison of IADs across groups and ages, a mixed model repeated measures ANOVA with Bonferroni correction was used. For comparison of non-parametric values (categories of ventricular volume), Wilcoxon rank sum test was performed. A Type I error of *p* < 0.05 was considered significant. Mean diffusivity ellipsoids were graphed with MATLAB, R2017b.

## Results

This model incorporates prenatal CNS injury from CAM, early postnatal IVH with syngeneic lysed RBCs, and neurological assessment for 3 weeks through P21 in rodents, a period with some CNS developmental equivalence in humans to early childhood (**Figure 1A**). An initial cohort of 135 pups of both sexes was used to characterize a model of PHHP [43 sham-veh (sterile saline) intraventricular injection, 35 sham-IVH, 27 CAM-veh, and 30 CAM-IVH]. Presence of IVH after injection was confirmed by HUS in a subset of pups (**Figure 1B**). Early mortality (<48 h) did not differ between injection groups, and was 13% (18/135). No rats from the three control groups (sham-veh, sham-IVH and CAM-veh) became symptomatic from injections, while 4/24 (17%) of CAM-IVH rats that initially survived required euthanasia prior to their targeted endpoint due to progressive macrocephaly accompanied by lethargy and spastic movements. Ventriculomegaly was confirmed on post-mortem examination, and these animals were excluded from further analyses. Surviving rats (113) were collected on P5, P15, and P21.

### CAM-IVH Rats Exhibited Progressive Macrocephaly and Ventriculomegaly

In human infants, symptomatic PHHP is characterized by progressive macrocephaly with the head circumference crossing percentiles for corrected-age. By P7, CAM-IVH pups developed a dome-shaped cranium (**Figure 1C**). To track progressive macrocephaly, intra-aural distance (IAD) was measured daily as a surrogate for human head circumference. Beginning at P5, a significant, progressive increase in IADs was observed in CAM-IVH rats (*n* = 22) through P21, compared to sham-veh (*n* = 33, mixed model repeated measures with Bonferroni’s correction, *p* < 0.001), sham-IVH (*n* = 20, *p* < 0.001) and CAM-veh rats (*n* = 19, *p* < 0.001) (**Figure 1D**). Both sexes with CAM-IVH exhibited similar patterns of macrocephaly (**Supplementary Figures S1A,B**). Thus, after the combination of perinatal CAM-IVH, neonatal rats displayed progressive macrocephaly through P21, a key component of PHHP.

To quantify early changes in ventricular size, P5 HE-stained coronal sections were analyzed. Ventriculomegaly was observed in CAM-IVH rats (*n* = 9), but not in sham-veh (*n* = 8, two-way ANOVA with Bonferroni correction, *p* < 0.001) or sham-IVH (*n* = 4, *p* = 0.003) rats (**Figures 1E,F**). Consistent with our prior work (Jantzie et al., 2014a), CAM-veh rats (*n* = 5) showed moderate ventricular enlargement compared to shams, but smaller than CAM-IVH (all *p* > 0.05). Notably, the sham-IVH and CAM-veh rats with mild ventriculomegaly did not exhibit the progressive macrocephaly that was present only in CAM-IVH rats. These results show prenatal CAM plus postnatal IVH causes early ventriculomegaly.

To clarify whether the ventriculomegaly observed at P5 was transient or sustained, ventricular volumes were analyzed at P21 using *ex vivo* MRI. Ventricular volumes from CAM-IVH rats (*n* = 8) were significantly larger than sham-veh (*n* = 5), sham-IVH rats (*n* = 4), and CAM-veh rats (*n* = 6, **Figure 1G**). Ventricular volume at P21 correlated with macrocephaly (Pearson correlation *r* = 0.338, *p* = 0.012, **Table 1**). Together, IAD measurements and ventricular volumes from histology and MRI demonstrate that only the combination of prenatal CAM plus early postnatal IVH leads to progressive macrocephaly with persistent ventriculomegaly, characteristic of PHHP.

**Table 1:** Pearson *r* correlation of phenotypic parameters with significant DTI scalars.

Parameter				Cliff aversion		Ventricular volume	

				Pearson *r*	*p*-value	Pearson *r*	*p*-value
Macrocephaly (P21 IAD) Cliff aversion				0.281 X	***p* = 0.040**	0.338 0.461	***p* = 0.012** ***p* < 0.001**

**DTI scalars**		**Macrocephaly P21 IAD**		**Cliff aversion**		**Ventricular volume**	
		**Pearson *r***	***p*-value**	**Pearson *r***	***p-*value**	**Pearson *r***	***p-*value**

White matter	FA	-0.585	***p* = 0.001**	-0.447	***p* = 0.012**	-0.376	***p* = 0.040**
Corpus callosum	FA	-0.427	***p* = 0.015**	-0.361	***p* = 0.006**	-0.325	*p* = 0.070
Hippocampus	FA	-0.541	***p* = 0.001**	-0.247	*p* = 0.181	-0.099	*p* = 0.591
Striatum	FA	-0.318	*p* = 0.076	-0.444	***p* = 0.012**	-0.205	*p* = 0.261
Thalamus	FA	-0.201	*p* = 0.270	-0.320	*p* = 0.079	-0.210	*p* = 0.249
White matter	MD	0.422	***p* = 0.016**	0.320	*p* = 0.079	0.291	*p* = 0.106
Corpus callosum	MD	0.467	***p* = 0.007**	0.382	***p* = 0.034**	0.383	***p* = 0.030**
White matter	RD	0.435	***p* = 0.013**	0.385	***p* = 0.033**	0.619	***p* < 0.000**
Corpus callosum	RD	0.552	***p* = 0.001**	0.350	***p* = 0.006**	0.445	***p* = 0.011**

*Bolded values indicate statistical significance.*

### Cumulative Insults Precipitated Developmental Delay

To assess neonatal neurodevelopment, a battery of age-appropriate, validated, functional tests (Farkas et al., 2009; Baharnoori et al., 2012) was administered. Most responses, including somatic reflexes such as eye opening and startle response, did not vary amongst the four groups (**Supplementary Figure S2**). The forelimb placement reflex, the placement of the forelimb on a shelf in response to stroking the dorsum of the forelimb while the rat is suspended near the shelf, was worse in CAM-veh and CAM-IVH rats compared to sham-veh rats (**Supplementary Figure S2G**). Cliff aversion, the time it takes a pup placed with its head and forelimbs over a ledge to return to a safe position, was longer in injured rats, consistent with early developmental delay. Specifically, CAM-IVH rats (*n* = 24) exhibited longer cliff aversion times compared to sham-veh rats (*n* = 24, *p* = 0.002), and sham-IVH rats (*n* = 18, *p* < 0.001, **Figure 1H**). The CAM-veh rats (*n* = 31) also showed impairment with cliff aversion, but did not differ significantly from sham-veh or CAM-IVH rats. The pattern of poor cliff aversion performance by CAM-IVH rats was more pronounced in male rats than female rats (**Supplementary Figures S1E,F**). Notably, cliff aversion performance correlated with macrocephaly (*r* = 0.281, *p* = 0.040), and more robustly with ventriculomegaly (*r* = 0.461, *p* < 0.001, **Table 1**), suggesting that in this model early functional outcomes are associated with macrocephaly and ventriculomegaly.

### Correlation of Phenotype With Early CNS Insults

To estimate the relative contribution of early CNS insults to phenotypic parameters important for PHHP, correlations were tested between individual findings and types of CNS insults. Macrocephaly (P21 IAD) correlated only with the combination of CAM-IVH (*r* = 0.420, *p* = 0.010), and not with CAM or IVH in isolation (**Table 2**). Similarly, ventriculomegaly correlated only with CAM-IVH (*r* = 0.765, *p* = 0.002), and not with CAM or IVH alone. Poor cliff aversion performance also correlated with CAM-IVH (*r* = 0.467, *p* = 0.004, **Table 2**). Overall, only the combined CAM-IVH correlated robustly with macrocephaly, ventriculomegaly, and poor functional performance that are characteristic of PHHP.

**Table 2:** Pearson *r* correlation of CNS insults with phenotypic parameters and significant DTI scalars.

Clinical parameter	Chorioamnionitis	IVH	Chorioamnionitis plus IVH (vehicle)^∗^	Chorioamnionitis plus IVH (EPO+MLT)^∗∗^
	Pearson *r p*-value	Pearson *r p*-value	Pearson *r p*-value	Pearson *r p*-value
Macrocephaly (P21 IAM)	-0.103 *p* = 0.574	-0.192 *p* = 0.405	0.420 ***p* = 0.010**	-0.599 ***p* < 0.001**
Cliff aversion	0.323 *p* = 0.062	-0.462 *p* = 0.015	0.467 ***p* = 0.004**	-0.367 ***p* = 0.042**
Ventricular volume	0.522 *p* = 0.100	-0.028 *p* = 0.943	0.765 ***p* = 0.002**	-0.341 *p* = 0.056

**DTI scalar**	**Chorioamnionitis**	**IVH**	**Chorioamnionitis plus IVH (vehicle)**^∗^	**Chorioamnionitis** **plus IVH (EPO+MLT)**^∗∗^
	**Pearson *r p*-value**	**Pearson *r p-*value**	**Pearson *rp*-value**	**Pearson *rp*-value**

White matter FA	-0.421 *p* = 0.197	-0.626 *p* = 0.071	-0.813 ***p* = 0.002**	0.800 ***p* < 0.001**
Corpus callosum FA	-0.532 *p* = 0.092	-0.374 *p* = 0.322	-0.791 ***p* = 0.001**	0.830 ***p* < 0.001**
Hippocampus FA	-0.266 *p* = 0.430	0.122 *p* = 0.773	-0.735 ***p* = 0.004**	0743 ***p* = 0.001**
Striatum FA	-0.411 *p* = 0.209	-0.663 *p* = 0.052	-0.641 ***p* = 0.018**	0.801 ***p* < 0.001**
Thalamus FA	-0.637 ***p* = 0.035**	0.244 *p* = 0.527	-0.640 ***p* = 0.019**	0.896 ***p* = 0.012**
White matter MD	0.261 *p* = 0.437	0.176 *p* = 0.651	0.792 ***p* = 0.001**	-0.260 *p* = 0.313
Corpus callosum MD	0.356 *p* = 0.282	0.046 *p* = 0.927	0.836 ***p* < 0.001**	-0.670 ***p* = 0.003**
White matter RD	0.391 *p* = 0.234	0.392 *p* = 0.296	0.874 ***p* < 0.001**	-0.347 *p* = 0.173
Corpus callosum RD	0.268 *p* = 0.425	0.036 *p* = 0.927	0.863 ***p* < 0.001**	-0.876 ***p* < 0.001**

*Bolded values indicate statistical significance. ^∗^Chorioamnionitis, IVH and CAM-IVH rat correlations compared with sham-veh rats. ^∗∗^EPO+MLT-treated CAM-IVH rats compared with vehicle-treated CAM-IVH rats.*

### EPO+MLT Prevented Macrocephaly and Neurodevelopmental Delay in PHHP-Like Rats

**FIGURE 2:**
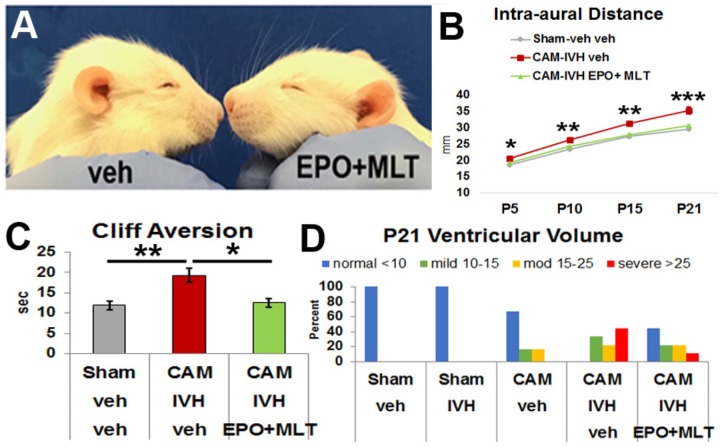
Extended neonatal treatment with erythropoietin (EPO) plus melatonin (MLT) prevents progressive macrocephaly and neurodevelopmental delay, and reduces sustained ventriculomegaly in CAM-IVH rats. **(A)** At P16, a vehicle-treated CAM-IVH rat exhibits a domed cranial vault, while the EPO+MLT-treated CAM-IVH rat does not. **(B)** IADs of EPO+MLT-treated CAM-IVH rats diverge from those of vehicle-treated CAM-IVH rats, and become similar to sham-veh rats. **(C)** EPO+MLT treatment of CAM-IVH rats prevents delayed performance on cliff aversion, compared to vehicle-treated CAM-IVH rats. **(D)** Ventricular volume measured on MRI at P21 was defined as normal, and mild, moderate and severe ventriculomegaly. Sham-veh and sham-IVH rats all had normal size ventricles, while all vehicle-treated CAM-IVH rats had ventriculomegaly. After neonatal EPO+MLT treatment, 40% of CAM-IVH rats had normal size ventricles, and the proportion with more severe ventriculomegaly shifted to less severe. Importantly, none of the EPO+MLT CAM-IVH rats with residual ventriculomegaly had macrocephaly. (**B**: a mixed model repeated measures ANOVA with Bonferroni’s correction; **C**: two-way ANOVA with Bonferroni’s correction; **D**: Wilcoxon rank sum test, ^∗^*p* < 0.05, ^∗∗^*p* ≤ 0.01, ^∗∗∗^*p* ≤ 0.001.)

To determine if the progressive macrocephaly and poor cliff aversion performance observed in CAM-IVH rats was preventable, we assessed the efficacy of combined EPO+MLT treatment in the neonatal period in a separate cohort of rats (*n* = 88). Specifically, on P2, CAM-IVH rats were randomized to an extended regimen of EPO+MLT or vehicle from P2 to P10. To determine whether the combination of EPO+MLT had any unanticipated impact on neurodevelopment, naïve rats were randomized to the same EPO+MLT regimen (*n* = 20), or vehicle (*n* = 10). No differences in neurodevelopment were detected (**Supplementary Figure S3**), consistent with prior studies showing no detrimental effects of EPO or MLT in shams (Mazur et al., 2010; Carloni et al., 2014, 2016). Thus, shams received only vehicle for the remainder of the studies. While vehicle-treated CAM-IVH rats exhibited a domed head, EPO+MLT-treated CAM-IVH rats did not (**Figure 2A**). At P5, the IADs of the EPO+MLT-treated CAM-IVH rats (*n* = 15) were significantly lower than IADs of the vehicle-treated CAM-IVH rats (*n* = 28, mixed model repeated measures with Bonferroni correction, *p* = 0.017, **Figure 2B**). By P21, the IADs of the EPO+MLT-treated rats remained similar to shams (*n* = 28), and were markedly less than the vehicle-treated CAM-IVH rats (*p* < 0.001). By contrast, when EPO alone (*n* = 9), or MLT alone (*n* = 5) were administered to CAM-IVH rats, the IADs were not significantly different compared littermates that received vehicle (*n* = 8, **Supplementary Figure S1I**). The EPO+MLT treatment prevented progressive macrocephaly in both male and female rats (**Supplementary Figures S1C,D**). In addition to preventing macrocephaly, neonatal EPO+MLT treatment also mitigated poor performance on cliff aversion after CAM-IVH. Compared to vehicle-treated CAM-IVH rats (*n* = 24), EPO+MLT-treated CAM-IVH rats (*n* = 17) exhibited shorter cliff aversion times (*p* = 0.026), and performed similar to shams (*n* = 24, **Figure 2C**). None of the other reflexes were significantly impacted by EPO+MLT, although a trend for surface righting was apparent (**Supplementary Figure S2**). While CAM-IVH rats of both sexes showed a similar pattern of improved cliff aversion performance with neonatal EPO+MLT treatment, the differences were more prominent in males (**Supplementary Figures S1G,H**). Thus, neonatal EPO+MLT treatment prevented progressive macrocephaly and early signs of developmental delay exhibited by vehicle-treated CAM-IVH rats.

### EPO+MLT Treatment Improved Ventriculomegaly in PHHP-Like Rats

To determine if EPO+MLT treatment impacted ventriculomegaly, we evaluated ventricular volume at P21. Ventricular volume from *ex vivo* MRI was classified *a priori* as normal (<10 mm^3^), mild (10–<15 mm^3^), moderate (15–25 mm^3^) or severe (>25 mm^3^) ventriculomegaly (**Figure 2D**). All of the sham-veh (5/5) and sham-IVH (4/4) rats had normal ventricular volume. Two-thirds (4/6) of CAM-veh rats exhibited a normal volume, while one-third had mild or moderate ventriculomegaly without macrocephaly, consistent with posthemorrhagic ventricular dilation observed in some toddlers born preterm with CNS injury (Pappas et al., 2018). All (9/9) of the vehicle-treated CAM-IVH rats showed ventriculomegaly (33% mild, 22% moderate, and 44% severe). By contrast, 44% (4/9) of EPO+MLT-treated rats had a normal ventricular volume and only 11% had severe ventriculomegaly, a significant reduction in the proportion with ventriculomegaly compared to vehicle-treated CAM-IVH rats (Wilcoxon rank sum test, *p* = 0.005). The reduction of ventriculomegaly combined with the lack of macrocephaly suggests that PHHP occurs along a spectrum of severity that can be modulated with a timely neuroreparative intervention.

### Microstructural Abnormalities Improved With EPO+MLT Treatment

**FIGURE 3:**
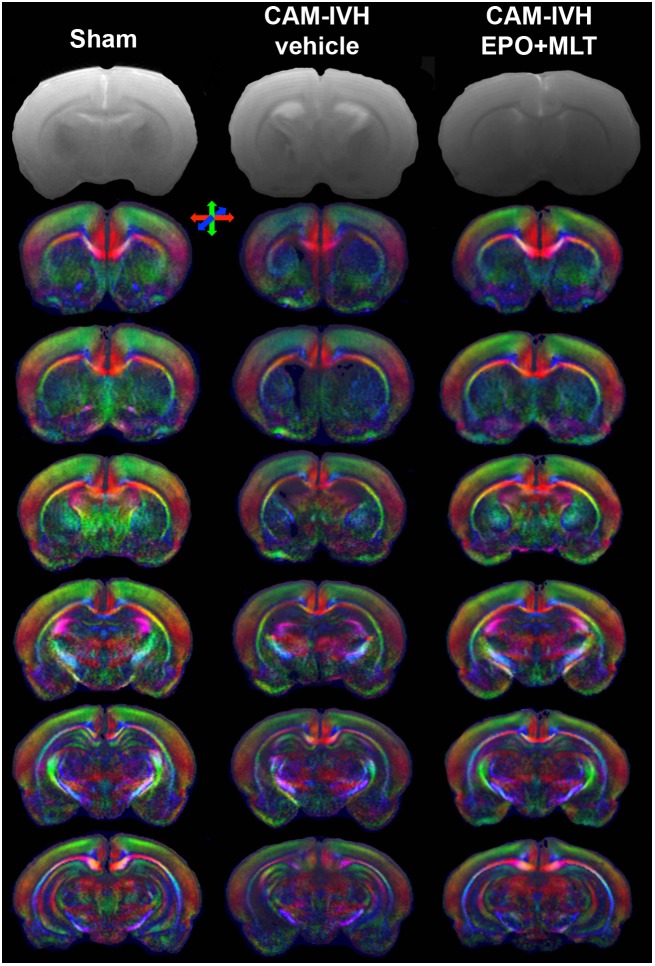
Coronal T2 images demonstrate ventricular size. Directionally encoded color maps of coronal images reveal widespread white and gray matter microstructural abnormalities in vehicle-treated CAM-IVH rats that are prevented by neonatal EPO+MLT treatment. Color maps show loss of microstructural coherence and impaired directional diffusion in regions with poor microstructural integrity. Directional colors are red for transverse, green for vertical and blue for orthogonal to the plane.

**FIGURE 4:**
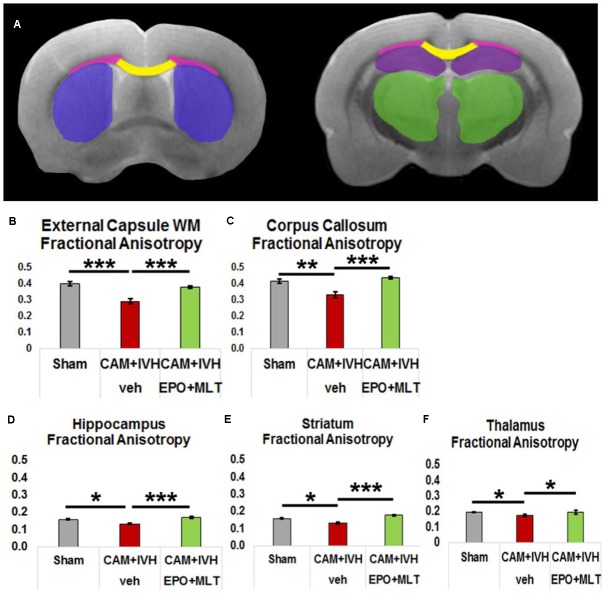
Diffusion tensor imaging (DTI) regional analyses and fractional anisotropy (FA) graphs quantify prevention of loss of microstructural integrity. **(A)** Diagram of DTI regions of interest (purple – striatum, yellow – corpus callosum, pink – external capsule white matter (ECWM), green – thalamus, and maroon – hippocampus). **(B)** Reduction of FA in the ECWM of vehicle-treated CAM-IVH rats is prevented in CAM-IVH rats with neonatal EPO+MLT treatment. **(C)** Similarly, reduction of FA in the central corpus callosum of vehicle-treated CAM-IVH rats is prevented by EPO+MLT treatment. **(D)** FA in the hippocampus is also decreased in vehicle-treated CAM-IVH rats compared to shams, and EPO+MLT treatment mitigates the damage. **(E)** Likewise, FA in the striatum is reduced in vehicle-treated CAM-IVH rats, and prevented in EPO+MLT-treated CAM-IVH rats. **(F)** FA in the thalamus is also lower in vehicle-treated CAM-IVH rats compared to shams, and normalizes with EPO+MLT treatment (all comparisons are two-way ANOVA with Bonferroni’s correction, ^∗^*p* < 0.05, ^∗∗^*p* ≤ 0.01, ^∗∗∗^*p* ≤ 0.001).

Due to the challenges in obtaining reliable functional outcomes in neonates, DTI has emerged as an imaging biomarker for perinatal brain injury (Vollmer et al., 2017; Hollund et al., 2018; Olsen et al., 2018). In particular, congenital hydrocephalus in children is associated with widespread DTI microstructural abnormalities (Mangano et al., 2016; Zhao et al., 2016). To supplement the functional outcome quantified with the behavior battery, we assessed regional variation in white and gray matter microstructure (**Figures 3**, **4A**). Vehicle-treated CAM-IVH rats (*n* = 8) had markedly lower external capsule white matter (ECWM) fractional anisotropy (FA) at P21 compared to shams (*n* = 5, two-way ANOVA with Bonferroni’s correction *p* < 0.001), that improved with EPO+MLT (*n* = 9, *p* = 0.001, **Figure 4B**) A similar pattern with FA was observed in the central corpus callosum (shams versus vehicle-treated CAM-IVH, *p* = 0.002; vehicle-treated versus EPO+MLT-treated CAM-IVH, *p* < 0.001, **Figure 4C**). Likewise, gray matter FA was lower in vehicle-treated CAM-IVH rats compared to shams in the hippocampus (*p* = 0.022, **Figure 4D**), striatum (*p* = 0.028, **Figure 4E**) and thalamus (*p* = 0.030, **Figure 4F**). Significantly, neonatal EPO+MLT treatment in CAM-IVH rats prevented loss of gray matter microstructural integrity in hippocampus (*p* < 0.001), striatum (*p* < 0.001), and thalamus (*p* = 0.018). Thus, while CAM-IVH causes extensive loss of microstructural integrity by P21, neonatal EPO+MLT treatment prevents this widespread damage.

**FIGURE 5:**
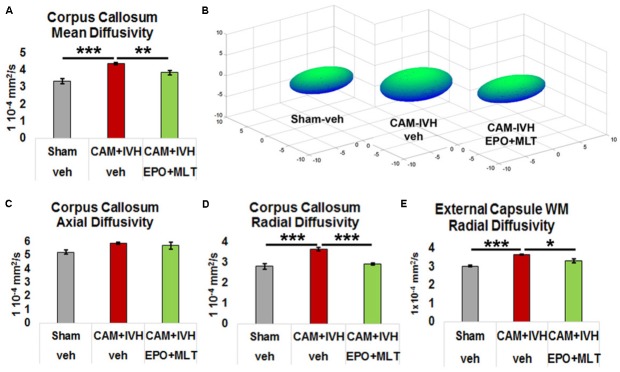
Directional diffusivity in central corpus callosum and external capsule white matter is altered by CAM-IVH and EPO+MLT treatment. **(A)** Mean diffusivity is elevated in the corpus callosum vehicle-treated CAM-IVH rats, compared to shams and EPO+MLT-treated CAM-IVH rats. **(B)** Ellipsoids comprised of the three major eigenvectors of diffusion in the central corpus callosum illustrate the changes in vehicle-treated and EPO+MLT-treated CAM-IVH rats. **(C)** Axial diffusivity is relatively unaffected by CAM-IVH. **(D)** Vehicle-treated CAM-IVH rats exhibit elevated radial diffusivity (RD) in central corpus callosum, and neonatal EPO+MLT treatment normalizes the microstructural integrity. **(E)** Similarly, vehicle-treated CAM-IVH rats show increased RD in the ECWM, and neonatal EPO+MLT treatment normalizes the RD (all comparisons are two-way ANOVA with Bonferroni’s correction, ^∗^*p* < 0.05, ^∗∗^*p* ≤ 0.01, ^∗∗∗^*p* ≤ 0.001).

To more specifically examine microstructural integrity in the corpus callosum and ECWM, we assessed directional diffusion. Mean diffusivity (MD) was elevated in the corpus callosum of vehicle-treated CAM-IVH rats compared to shams (*p* = 0.001) and EPO+MLT-treated CAM-IVH rats (*p* = 0.008, **Figures 5A,B**). Axial diffusivity was not altered in the corpus callosum of CAM-IVH rats (**Figure 5C**). By contrast, radial diffusivity (RD), a potential imaging biomarker of myelin injury, was also elevated in the corpus callosum of vehicle-treated CAM-IVH rats, compared to shams and EPO+MLT-treated CAM-IVH rats (both *p* < 0.001, **Figure 5D**). Similarly, ECWM RD was elevated in vehicle-treated CAM-IVH rats, compared to shams (*p* = 0.001) and EPO+MLT-treated CAM-IVH rats (*p* = 0.027, **Figure 5E**). Together, these findings show that the corpus callosum and ECWM are particularly vulnerable to CAM-IVH, and that early EPO+MLT treatment can prevent microstructural abnormalities caused by prenatal CAM plus IVH.

We tested correlations of DTI scalars with macrocephaly, impaired cliff aversion performance, and ventriculomegaly (**Table 1**). Macrocephaly (P21 IAD) correlated robustly with FA in the ECWM (*p* = 0.001), corpus callosum (*p* = 0.015) and hippocampus (*p* = 0.001). Similarly, poor cliff aversion performance correlated strongly with FA in the ECWM (*p* = 0.012), corpus callosum (*p* = 0.006), and striatum (*p* = 0.012), while ventriculomegaly correlated with corpus callosum FA (*p* = 0.04). For mean diffusivity, all three phenotypic metrics (macrocephaly, poor cliff aversion performance, and ventriculomegaly) showed strong correlation with corpus callosum MD (all *p* < 0.03, **Table 1**), while only macrocephaly was associated with altered ECWM MD (*p* = 0.016). Moreover, for radial diffusivity, all three phenotypic metrics showed strong correlation with both corpus callosum and ECWM RD (all *p* < 0.035, **Table 1**). Together, these finding show DTI scalars, particularly RD of white matter, are indicative of phenotypic parameters that most closely resemble PHHP, and suggest that metrics of directional diffusion may serve as biomarkers of PHHP severity and recovery.

Next, to test the impact of different components of CNS injury on DTI scalars, we examined the correlations between CAM, IVH, and CAM-IVH compared to shams. Only thalamic FA correlated with CAM alone (*p* = 0.035), and only striatal FA showed a trend with IVH alone (*p* = 0.052, **Table 2**). By contrast, FA from both white and gray matter correlated with CAM-IVH (all *p* < 0.02). Moreover, for both ECWM and corpus callosum, both MD and RD showed very robust correlations with CAM-IVH (all *p* ≤ 0.001). Finally, the correlations between DTI scalars for vehicle-treated compared to EPO+MLT-treated CAM-IVH rats were analyzed. These also showed strong correlations with DTI scalars when compared with vehicle-treated CAM-IVH rats (**Table 2**). Thus, in addition to DTI correlating with phenotypic parameters, DTI scalars may hold promise as imaging biomarkers for specific CNS insults associated with PHHP, and neurorepair.

### EPO+MLT Treatment Ameliorated Morphological Damage to Ependymal Motile Cilia

**FIGURE 6:**
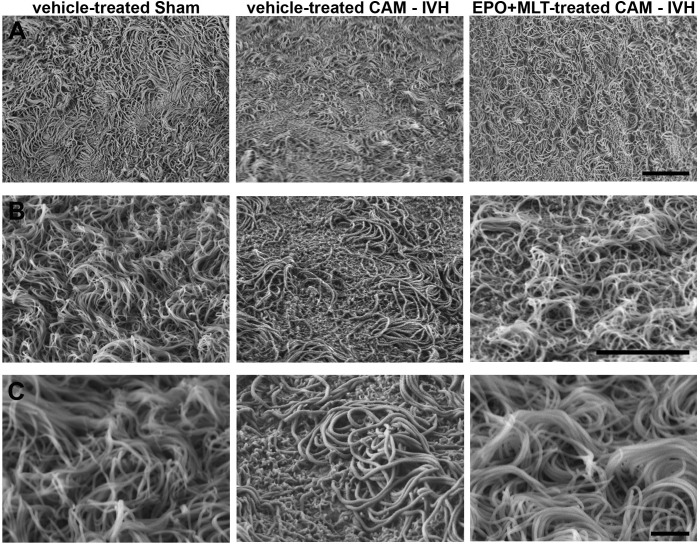
Scanning electron microscopy (SEM) images of P21 ependymal motile cilia show preservation of cilia following EPO+MLT treatment in CAM-IVH rats. **(A)** Low magnification images demonstrate a sheet of tuft-like morphology in sham-veh rats that is disrupted in vehicle-treated CAM-IVH rats. CAM-IVH rats with EPO+MLT treatment exhibit preservation of the sheet of tufts of motile cilia. Bar = 10 microns. **(B)** Higher magnification images reveal some motile cilia in vehicle-treated CAM-IVH rats are missing. Bar = 10 microns. **(C)** High magnification images show the residual cilia appear bloated and matted in vehicle-treated rats, unlike the thinner, more upright cilia organized in tufts in sham-veh rats or EPO+MLT-treated CAM-IVH rats. Bar = 2 microns.

To examine EMC morphology, scanning electron microscopy (SEM) was performed on anatomically matched micro-dissected portions of the lateral ventricular wall on P21. Cilia from sham rats appeared in organized tufts (**Figure 6**). By contrast, a subset of cilia from vehicle-treated CAM-IVH rats were missing, while the remaining cilia were matted, flattened, and lacked the tuft-like appearance found in shams. Importantly, EPO+MLT treatment prevented the damage and restored the tuft-like appearance of EMC in CAM-IVH rats. Thus, these results demonstrate neonatal EPO+MLT treatment can modulate structural damage to EMC after CAM-IVH.

### EPO+MLT Prevented Low Yes-Associated Protein (YAP) mRNA Levels at P15

**FIGURE 7:**
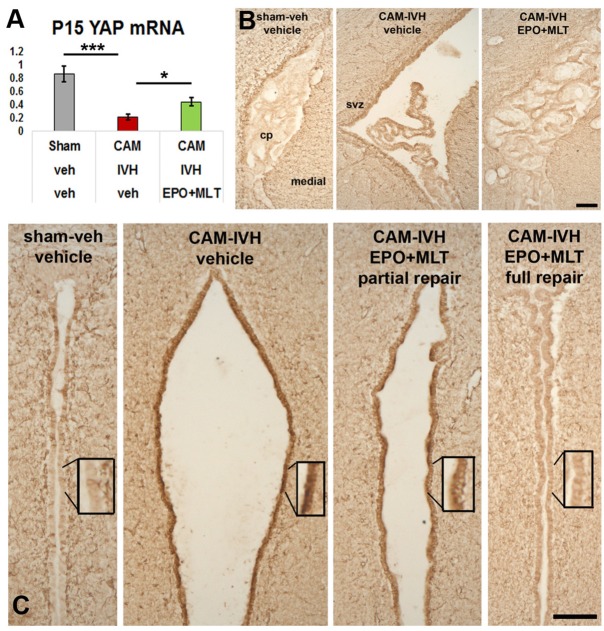
Neonatal EPO+MLT treatment in CAM-IVH rats improves periventricular yes-associated protein (YAP) mRNA levels and reduces GFAP-expression. **(A)** At P15, periventricular YAP mRNA levels are reduced in micro-dissected ependyma from vehicle-treated CAM-IVH rats, compared to shams. This loss is partially, but significantly, prevented in CAM-IVH rats following EPO+MLT treatment. **(B)** GFAP-immunolabeling of coronal sections of the lateral ventricle show the choroid plexus appears shrunken in the large ventricle. EPO-MLT-treatment prevents this appearance of the choroid plexus. **(C)** Minimal GFAP-immunolabeling is present in the normal caliber third ventricle of a sham rat, while marked GFAP expression is present in the ependymal lining of the dilated third ventricle in a vehicle-treated CAM-IVH rat. In an EPO+MLT-treated CAM-IVH rat with mild ventriculomegaly, there is less GFAP present, and this pattern is more apparent in the EPO+MLT-treated CAM-IVH rat with a normal sized third ventricle (Bars = 100 microns, two-way ANOVA with Bonferroni’s correction, ^∗^*p* < 0.05, ^∗∗∗^*p* ≤ 0.001).

Next, we tested whether EPO+MLT treatment impacted YAP mRNA transcription levels in micro-dissected ependyma at P15. Reduction of YAP mRNA levels occurred in vehicle-treated CAM-IVH rats (*n* = 4), compared to shams (*n* = 7, *p* ≤ 0.001, **Figure 7A**). Neonatal EPO+MLT treatment following CAM-IVH (*n* = 5) mitigated loss of YAP mRNA, compared to vehicle treated CAM-IVH (two-way ANOVA with Bonferroni correction, *p* = 0.036). These results provide initial support for our hypothesis that CAM-IVH reduces YAP transcription, and that EPO+MLT treatment prevents loss of YAP mRNA.

### EPO+MLT Treatment Reduced Excess Ependymal GFAP Expression

To determine whether CAM-IVH substantially altered periventricular gliosis, P21 coronal sections were immunolabeled with GFAP (glial fibrillary acidic protein) antibodies. In the lateral ventricle, GFAP-immunolabeling in vehicle-treated CAM-IVH rats differed from shams, and demonstrated changes in both the ependyma and choroid plexus (**Figure 7B**). After EPO+MLT treatment, the ependyma showed less GFAP+ reactivity, and the choroid plexus resembled the choroid plexus of shams. Ependymal changes were more dramatic in the third ventricle (**Figure 7C**), consistent with a diffuse ependymal reaction after CAM-IVH. Sham rats showed no enlargement of the third ventricle, and minimal GFAP+ cells in the ependymal lining (**Figure 7C**). By contrast, ependyma in vehicle-treated CAM-IVH rats with severe ventriculomegaly exhibited dense GFAP+ expression, consistent with ventricular disruption observed in human infants with IVH (McAllister et al., 2017). GFAP-immunolabeling was moderate in EPO+MLT-treated CAM-IVH rats with residual mild ventriculomegaly, while EPO+MLT-treated rats with normal ventricular size had minimal GFAP-immunolabeling, similar to shams. Notably, in this age-appropriate CAM-IVH model, the degree of GFAP expression appeared to vary inversely with ventriculomegaly.

## Discussion

We developed a clinically relevant model of PHHP that exhibits essential components of PHHP, progressive macrocephaly with ventriculomegaly, and used this model to test whether extended EPO+MLT treatment can prevent the development of PHHP. We found that EPO+MLT prevented progressive macrocephaly and impaired cliff aversion performance, and reduced ventriculomegaly. Neonatal EPO+MLT treatment also normalized damage from CAM-IVH to microstructural integrity of white and gray matter, ultrastructural injury to EMC, periventricular YAP levels, and ependymal GFAP-immunolabeling. These data indicate that the combination of inflammation from prenatal CAM plus early postnatal IVH damages EMC, likely limiting propulsion of CSF and leading to symptomatic PHHP. The well-characterized prenatal injury model of CAM used here causes pathological and functional changes that mimic consequences of CNS injury from extremely preterm birth (Jantzie et al., 2014a, 2018; Maxwell et al., 2015). In juvenile rats, prenatal CAM produces a gait deficit reminiscent of the spastic gait of cerebral palsy (Jantzie et al., 2014a), and impaired executive function, social interaction and hyperactivity (Jantzie et al., 2018). Importantly, because of the staggered timing of CNS development and birth in rats compared to humans [birth in Sprague-Dawley rats is equivalent approximately to 30 weeks gestation in humans, and P7-P10 in the rat is equivalent to a term human (Semple et al., 2013; Jantzie and Robinson, 2015)], newborn rats mimic early preterm neonates and allow age-appropriate testing of interventions. This novel model of PHHP mimics the prenatal impact of CAM and intrauterine inflammation on CNS maturation, plus replicates the timing of IVH in early preterm infants by injecting lysed RBCs on P1. Pups exposed to prenatal CAM-IVH developed persistent ventriculomegaly with progressive macrocephaly, two hallmarks of PHHP, accompanied by histological, functional and imaging findings consistent with those observed in preterm infants with PHHP. EMC in CAM-IVH rats exhibited damage, similar to damaged EMC observed after intracerebral hematoma and TBI in the mature CNS (Xiong et al., 2014; Chen et al., 2015). The combination of macrocephaly with ventriculomegaly reported here is also similar to the morphology of models of symptomatic hydrocephalus resulting from genetic mutations that impede EMC development and/or function (Banizs et al., 2005; Tissir et al., 2010; Yung et al., 2011; Peng et al., 2013; Vidovic et al., 2015; Park et al., 2016; Muniz-Talavera and Schmidt, 2017; Nunez-Olle et al., 2017; Takagishi et al., 2017; Abdelhamed et al., 2018; Xu et al., 2018). Together, the unique combination of CAM plus IVH, along with the age-appropriate timing of both insults, increases the clinical relevance of this novel model.

We used this PHHP model to test whether systemic, extended high-dose neonatal treatment with EPO+MLT could promote recovery and prevent hydrocephalus. PHHP results from a complex pattern of cumulative insults over the perinatal period, and extended dosing with EPO+MLT likely normalizes the microenvironment and enhances recovery over a sustained critical neurodevelopmental window. Preterm infants have different clinical risks of CNS damage, arising from varying patterns of IVH distribution and severity, plus other forms of neonatal injury such as periventricular leukomalacia and sepsis, and the genetic predisposition that impacts the response to these cumulative insults. The extended dosing period and combinatorial therapies likely addresses the varying severity, timing and individual predisposition to damage better than using a single agent over the developmental window. Also, CAM and IVH both affect multiple cell types in widespread CNS regions, a complex amalgam that has been identified in other forms of CNS injury from preterm birth (Volpe, 2009). This diffuse, complex, cumulative pattern of injury sustained over several weeks by preterm infants differs substantially from a regionally and temporally specific injury such as a single occlusive middle cerebral artery stroke. EPO and MLT also have numerous overlapping complementary mechanisms of action on neural cells that result in repair (Brines et al., 2000; Carloni et al., 2008). Receptors for both EPO and MLT are present on neural cells (Mazur et al., 2010; Bahna and Niles, 2017; Ng et al., 2017; Osier et al., 2017; Tsai et al., 2017), and both EPO and MLT enhance the genesis, survival and maturation of multiple neural cell types (Iwai et al., 2010; Mazur et al., 2010; Jantzie et al., 2013; Li et al., 2017; Zhang et al., 2018). EPO and MLT reduce neuroinflammation and oxidative stress (Carloni et al., 2016; Ramirez-Jirano et al., 2016; Dominguez Rubio et al., 2017; McDougall et al., 2017; Wang et al., 2017; Wei et al., 2017; Zhou et al., 2017). They also restore the microenvironment by limiting excess calpain activity that promotes cell death and destroys molecules essential for neurodevelopment (Samantaray et al., 2008; Jantzie et al., 2014b, 2016; Robinson et al., 2016). EPO and MLT reduce endoplasmic reticulum stress and mitochondrial dysfunction that may propagate chronic damage and precipitate early neurodegeneration (Hong et al., 2012; Das et al., 2013; Carloni et al., 2014; Fernandez et al., 2015; Hadj Ayed Tka et al., 2015; Zhao et al., 2015; Hu et al., 2016; Hardeland, 2017; Mendivil-Perez et al., 2017; Xue et al., 2017). Additionally, EPO+MLT treatment likely suppresses LPA, increases YAP, and enhances ependymal renewal, which when combined with white matter repair, provides a unique advantage to stimulate recovery from the cascade of insults the cumulatively lead to PHHP. Further work to delineate the specific mechanisms of how EPO and MLT act together to optimize EMC maturation following CAM-IVH are underway.

Systemic inflammation, particularly from maternal causes, is associated with a higher risk of IVH (Moscuzza et al., 2011; Salas et al., 2013; Arayici et al., 2014; Shankaran et al., 2014; Lee et al., 2016; Lu et al., 2016; Stark et al., 2016; Chevallier et al., 2017; Edwards et al., 2018). The Gram-negative endotoxin LPS used here binds TLR4 (toll-like receptor 4), a receptor implicated in neonatal parenchymal microhemorrhages (Carusillo Theriault et al., 2017). Others have also shown that a TLR4 antagonist limits damage from intracerebral hemorrhage (Wang et al., 2013; Kwon et al., 2015). Systemic inflammation mediated via TLR4 increases choroid plexus CSF secretion (Karimy et al., 2017), and likely contributes to ventriculomegaly in hydrocephalus. We found that EPO+MLT treatment not only limited ependymal GFAP-expression, but also prevented altered morphology of the choroid plexus. Thus, there are multiple potential mechanisms to explain why this combinatorial therapy limits the development of PHHP.

We used this novel PHHP model to test the potential reversibility of ependymal injury. Based on prior work (Yung et al., 2011; Huang et al., 2016; Park et al., 2016), we reasoned that one potential explanation connecting systemic inflammation to the propensity to develop PHHP after IVH is that systemic inflammation from LPS impacts ependymal cells, and potentially neural cell progenitor differentiation, through alterations in periventricular YAP mRNA levels. Here we report that CAM-IVH reduces YAP transcription in micro-dissected ependyma, and that neonatal EPO+MLT prevents loss of YAP mRNA. These data are consistent with a role for YAP in the mitigation of EMC damage, as observed with SEM in this report.

Studies using DTI in children with hydrocephalus have shown alterations in white matter microstructural integrity (Mangano et al., 2016; Zhao et al., 2016), similar to what we have shown here in toddler-equivalent P21 vehicle-treated CAM-IVH rats. In preclinical studies, extended neonatal EPO treatment mitigated widespread DTI abnormalities in white and gray matter (Robinson et al., 2016, 2017). Here, we showed that sustained neonatal treatment with EPO+MLT normalizes subacute DTI changes at 3 weeks after CAM-IVH. To our knowledge, this is the first report of the prevention of DTI microstructural damage in a preclinical model of PHHP. Aojula et al. (2016) used the transforming growth factors-β antagonist decorin to prevent DTI changes at 2 weeks in a kaolin model of hydrocephalus. Similarly, neonatal kaolin resulted in DTI abnormalities at 5 and 10 days (Yuan et al., 2010). The DTI analyses here demonstrated that both white matter and gray matter, and particularly the corpus callosum, are sensitive to CAM-IVH. While a component of white matter vulnerability may be related to direct structural injury from ventriculomegaly, it is likely that myelination and EMC function are precise, highly regulated, relatively vulnerable processes with demanding energy expenditures and environmental requirements. We reasoned that the precise regulation of EMC maturation and function, as well as recovery of more widespread injury to white and gray matter after CAM-IVH, required a potent, multipronged intervention. This led us to test a neonatal, sustained, high-dose, neuroprotective, clinically relevant dosing regimen of EPO+MLT.

While clinical studies have shown that isolated neonatal symptomatic hydrocephalus does not cause chronic neurological deficits (Radic et al., 2015), children with PHHP often suffer from additional neurological co-morbidities such as cerebral palsy, behavioral abnormalities and impaired cognition. Prenatal inflammation plus postnatal inflammation increases the risk of white matter injury and spastic cerebral palsy (Yanni et al., 2017), and many preterm infants with IVH suffer from neonatal inflammation that likely contributes to their poor functional outcomes. A sustained EPO+MLT regimen, similar to the one used here, prevents chronic motor, social, behavioral and cognitive deficits in adult rats following prenatal CAM (Jantzie et al., 2018). In this study EPO+MLT treatment prevented poor performance on cliff aversion, however, tests of neonatal function in rodents are inherently limited. Using a strategy similar to EPO+MLT to induce multi-faceted repair, intravenous or intraventricular injection of umbilical-cord derived mesenchymal stromal cells on P6 improved early functional outcomes and reduced ventriculomegaly and GFAP-expression in rats with IVH on P4 or P5 (Ahn et al., 2013; Mukai et al., 2017). Repetitive dosing with EPO+MLT has the distinct advantages of providing sustained stabilization of the microenvironment over extended perinatal course, known safety profile, ease of administration, and cost. Early intervention for ventriculomegaly from IVH may improve long term cognition in very selected populations (Leijser et al., 2018), yet preterm infants with two or more surgeries as neonates have lower cognitive scores (Gano et al., 2015), and shunt surgery at a young age increases the risk of shunt malfunction (Riva-Cambrin et al., 2016). While the timing of interventions for sick preterm infants with PHHP is a complex and evolving controversy, non-surgical intervention such as neonatal EPO+MLT could potentially minimize the need for more invasive procedures and their associated risks.

The pathogenesis of PHHP is likely similar to other types of acquired hydrocephalus from TBI, adult IVH, subarachnoid hemorrhage, or meningitis. The association between CNS inflammation and hydrocephalus is well known (Del Bigio and Di Curzio, 2016). More recently the association with systemic inflammation has been found. Sepsis increases the risk of developing hydrocephalus in infants and young children with TBI (Rumalla et al., 2018). Similarly, Wessell et al. (2018) showed that sustained systemic inflammatory response syndrome (SIRS) increases the risk of developing shunt-dependent hydrocephalus after aneurysmal subarachnoid hemorrhage. Adults with severe TBI are more likely to need surgical treatment for hydrocephalus in the presence of ventriculitis/meningitis (Bauer et al., 2011; Hu et al., 2018). Interestingly, after adult stroke in a preclinical model, increased GFAP expression was found in the ependymal lining, and EMC were affected, but hydrocephalus did not develop (Young et al., 2013). Numerous differences in the manifestations of injury and potential for repair exist between the developing and mature CNS. Current preclinical experiments are underway to determine whether findings from this study are relevant to acquired hydrocephalus in adults.

This study has several limitations. Many important mechanistic questions are beyond the scope of this initial report. Additional experiments are needed to clarify the limits on the timing and extent of renewal of ependymal cells and EMC, and thus the duration of the optimal dosing regimen. While the results presented here support our hypothesis that EMC damage in the neonatal period can be modulated significantly by endogenous neuroprotective agents, the situation in human neonates with PHHP is likely more complex. More specifically targeted therapies may add benefit, particularly in vulnerable patients due to their genetic or epigenetic risk factors. Still, the findings presented here suggest that neonatal EPO+MLT may offer a safe, effective, cost-sensitive treatment for at least a subset of infants with PHHP. Additional studies are underway to test cognitive outcomes in young adult rats after CAM-IVH. This is exceedingly important as children with ventricular dilatation after IVH of prematurity have cognitive deficits (Holwerda et al., 2016), and those who require surgical intervention are at even higher risk for poor outcomes (Adams-Chapman et al., 2008; Holwerda et al., 2016). Elevated LPA levels from systemic inflammation may reduce YAP transcription and its specification of radial glial cells to differentiate into ependymal cells, however, investigation of this hypothesis requires additional studies to clarify the timing of damage, and therapeutic window for repair. Our initial findings suggest that ventriculomegaly in this model is associated with diffuse ependymal GFAP+ reaction, however, any potential causal relationship is currently unknown. Experiments to evaluate the long term impact of CAM-IVH and the association with macrocephaly and ventriculomegaly are underway. Moreover, while we demonstrated that both sexes develop progressive macrocephaly, the study was not sufficiently powered to determine how well each sex responds to EPO+MLT treatment. Despite these and other limitations, this novel model of PHHP and our initial findings using the clinically viable EPO+MLT treatment to prevent macrocephaly with ventriculomegaly, poor cliff aversion performance and associated microstructural abnormalities warrants additional investigation.

## Conclusion

In conclusion, early preterm birth and its associated complications, including IVH and PHHP, remain a serious global challenge to infant and childhood mortality and morbidity. The findings reported here address several of the needs identified in a recent NIH-sponsored symposium on hydrocephalus (McAllister et al., 2015). The age-appropriate, clinically relevant model of PHHP induced by prenatal CAM plus P1 IVH provides a means to test mechanisms and potential interventions to modulate hydrocephalus in neonates. Our results support the use of DTI as an imaging biomarker of injury and repair for PHHP. Most importantly, use of a clinically viable regimen of endogenous neuroprotective agents EPO and MLT to prevent PHHP suggests that safe, economically sound, non-surgical treatments for hydrocephalus may be possible to transform the care of preterm infants with severe IVH in the near future.

## Availability of Data and Materials

All data generated or analyzed during this study are included in this published article and its **Supplementary Figure** files.

## Ethics Statement

This study was carried out in accordance with the recommendations and approval of the Institutional Animal Care and Use Committee (IACUC) at the University of New Mexico Health Sciences Center, and Johns Hopkins University.

## Author Contributions

SR and LJ conceived the hypothesis, designed, supervised, and performed the experiments, analyzed and interpreted data, and wrote the manuscript. FC, AO, TY, JN, NED, CS, and JM designed and performed the experiments and edited the manuscript. SJ performed the experiments, analyzed the data, and edited the manuscript. FN designed the experiments, interpreted the data, and edited the manuscript.

## Conflict of Interest Statement

The authors declare that the research was conducted in the absence of any commercial or financial relationships that could be construed as a potential conflict of interest.

## Supplementary Material

FIGURE S1 Both male and female rats exhibit similar patterns of progressive macrocephaly and poor cliff performance after CAM-IVH, and respond similarly to EPO+MLT treatment. **(A)** Male CAM-IVH rats (*n* = 10) show significant macrocephaly from P10 through P21, compared to sham-veh (*n* = 17), sham-IVH (*n* = 9), and CAM-veh (*n* = 10) rats. **(B)** Similarly, female CAM-IVH rats (*n* = 12) have larger IADs compared to sham-veh (*n* = 16), sham-IVH (*n* = 11), and CAM-veh rats (*n* = 9). **(C)** Neonatal EPO+MLT treatment prevents progressive macrocephaly in male CAM-IVH rats (*n* = 9), compared to vehicle-treated CAM-IVH rats (*n* = 13). By P21, IADs of male EPO+MLT-treated rats are similar to male shams (*n* = 15). **(D)** Similarly, the IADs of female EPO+MLT-treated rats (*n* = 6) match female shams (*n* = 13), and diverge from female vehicle-treated CAM-IVH rats (*n* = 15). **(E)** For cliff aversion, male CAM-IVH rats (*n* = 14) perform worse than sham-veh rats (*n* = 12) and sham-IVH rats (*n* = 8), while CAM-veh rats (*n* = 17) also perform poorly. **(F)** Female CAM-veh (*n* = 10) and CAM-IVH rats (*n* = 14) show a similar pattern, compared to sham-veh (*n* = 12) and sham-IVH (*n* = 10) rats, but the differences are not statistically significant. **(G)** Male vehicle-treated CAM-IVH rats (*n* = 14) perform poorly on cliff aversion compared to sham-veh rats (*n* = 12) and EPO+MLT–treated CAM-IVH rats (*n* = 8). **(H)** While the trend for female vehicle-treated CAM-IVH rats (*n* = 10) compared to sham-veh (*n* = 12) and EPO+MLT-treated CAM-IVH rats (*n* = 9) is similar to the male rats, the differences are not statistically significant. **(I)** To test the efficacy of EPO alone or MLT alone, CAM-IVH littermates were treated with vehicle (*n* = 8), EPO alone (*n* = 9), MLT alone (*n* = 5), or EPO+MLT (*n* = 8), and compared to vehicle-treated CAM-veh rats (*n* = 13). P21 IADs of EPO-treated or MLT-treated CAM-IVH rats were not different than vehicle-treated CAM-IVH rats. By contrast, IADs of EPO+MLT-treated CAM-IVH rats were different than vehicle-treated CAM-IVH rats, and were similar to the CAM-veh controls. (Comparisons are **A–D**: a mixed model repeated measures ANOVA; **E–I**: two-way ANOVA with Bonferroni’s correction, ^∗^*p* < 0.05, ^∗∗^*p* ≤ 0.01, ^∗∗∗^*p* ≤ 0.001.)

FIGURE S2 Neurobehavioral tests in neonatal rats are minimally affected by CAM-IVH. **(A)** Surface righting, the sum of the time to turn from lying on the back to upright on feet during three trials from P7 to P9, was mildly impaired in CAM-IVH rats compared to sham-veh rats, but the difference was not significant. The negative geotaxis (**B**, sum of time to redirect when placed on an inclined plane from pointing head down, with maximum of 30 s), the presence of forelimb grasp on P8 **(C)**, the presence of audio startle on P13 **(D)**, the presence of eye opening on P14 **(E)**, and the presence of ear twitch to cotton wisp on P14 **(F)** all did not differ between groups. **(G)** The presence of forelimb placement to stimulus on the dorsum of the forelimb on P15 was reduced in injured rats. **(H)** The ability to turn upright after being dropped upside down above a cushioned surface on P12 did not differ between groups. **(I)** Surface righting improved mildly in EPO+MLT-treated CAM-IVH rats compared to vehicle-treated CAM = IVH rats, but the trend was not significant. **(J–P)** None of the other reflexes differed significantly between the vehicle and EPO+MLT-treated CAM-IVH rats [*n* = 16–31, two-way ANOVA with Bonferroni correction **(A,B,I,J)** or Kruskal–Wallis with Dunn’s *post hoc* correction **(C–H,K–P)**]. ^∗^*p* < 0.05, ^∗∗∗^*p* < 0.001.

FIGURE S3 Neurodevelopment was assessed in naïve rats treated with EPO+MLT (*n* = 20) or vehicle (*n* = 10). Surface righting on P5–P7 **(A)**, negative geotaxis on P8–P14 **(B)**, cliff aversion on P12–P13 **(C)**, forelimb grasp on P10–P11 **(D)**, ear twitch on P14 **(E)**, forelimb placement on P15 **(F)**, and air righting on P12 **(G)** did not differ between vehicle-treated and EPO+MLT-treated naïve rats.

## References

[B1] Abdelhamed Z. Vuong S. M. Hill L. Shula C. Timms A. Beier D. (2018). A mutation in Ccdc39 causes neonatal hydrocephalus with abnormal motile cilia development in mice. *Development* 145:dev154500. 10.1242/dev.154500 29317443 PMC5825874

[B2] Adams-Chapman I. Hansen N. I. Stoll B. J. Higgins R. Network N. R. (2008). Neurodevelopmental outcome of extremely low birth weight infants with posthemorrhagic hydrocephalus requiring shunt insertion. *Pediatrics* 121 e1167–e1177. 10.1542/peds.2007-0423 18390958 PMC2803352

[B3] Ahn S. Y. Chang Y. S. Sung D. K. Sung S. I. Yoo H. S. Lee J. H. (2013). Mesenchymal stem cells prevent hydrocephalus after severe intraventricular hemorrhage. *Stroke* 44 497–504. 10.1161/STROKEAHA.112.679092 23287782

[B4] Alan N. Manjila S. Minich N. Bass N. Cohen A. R. Walsh M. (2012). Reduced ventricular shunt rate in very preterm infants with severe intraventricular hemorrhage: an institutional experience. *J. Neurosurg. Pediatr.* 10 357–364. 10.3171/2012.7.PEDS11504 22938077

[B5] Aojula A. Botfield H. Mcallister J. P. Gonzalez A. M. Abdullah O. Logan A. (2016). Diffusion tensor imaging with direct cytopathological validation: characterisation of decorin treatment in experimental juvenile communicating hydrocephalus. *Fluids Barriers CNS* 13:9. 10.1186/s12987-016-0033-2 27246837 PMC4888658

[B6] Arayici S. Kadioglu Simsek G. Oncel M. Y. Eras Z. Canpolat F. E. Oguz S. S. (2014). The effect of histological chorioamnionitis on the short-term outcome of preterm infants =32 weeks: a single-center study. *J. Matern. Fetal Neonatal Med.* 27 1129–1133. 10.3109/14767058.2013.850668 24093223

[B7] Baharnoori M. Bhardwaj S. K. Srivastava L. K. (2012). Neonatal behavioral changes in rats with gestational exposure to lipopolysaccharide: a prenatal infection model for developmental neuropsychiatric disorders. *Schizophr. Bull.* 38 444–456. 10.1093/schbul/sbq098 20805287 PMC3329978

[B8] Bahna S. G. Niles L. P. (2017). Epigenetic regulation of melatonin receptors in neuropsychiatric disorders. *Br. J. Pharmacol.* 10.1111/bph.14058 [Epub ahead of print]. 28967098 PMC6057907

[B9] Banizs B. Pike M. M. Millican C. L. Ferguson W. B. Komlosi P. Sheetz J. (2005). Dysfunctional cilia lead to altered ependyma and choroid plexus function, and result in the formation of hydrocephalus. *Development* 132 5329–5339. 16284123 10.1242/dev.02153

[B10] Bauer D. F. Mcgwin G. Jr. Melton S. M. George R. L. Markert J. M. (2011). Risk factors for conversion to permanent ventricular shunt in patients receiving therapeutic ventriculostomy for traumatic brain injury. *Neurosurgery* 68 85–88. 10.1227/NEU.0b013e3181fd85f4 21099716

[B11] Blencowe H. Cousens S. Oestergaard M. Z. Chou D. Moller A. B. Narwal R. (2012). National, regional, and worldwide estimates of preterm birth rates in the year 2010 with time trends since 1990 for selected countries: a systematic analysis and implications. *Lancet* 379 2162–2172. 10.1016/S0140-6736(12)60820-4 22682464

[B12] Brines M. Ghezzi P. Keenan S. Agnello D. De Lanarolle N. Cerami C. (2000). Erythropoietin crosses the blood-brain barrier to protect against experimental brain injury. *Proc. Natl. Acad. Sci. U.S.A.* 97 10526–10531. 10984541 10.1073/pnas.97.19.10526 PMC27058

[B13] Carloni S. Albertini M. C. Galluzzi L. Buonocore G. Proietti F. Balduini W. (2014). Melatonin reduces endoplasmic reticulum stress and preserves sirtuin 1 expression in neuronal cells of newborn rats after hypoxia-ischemia. *J. Pineal Res.* 57 192–199. 10.1111/jpi.12156 24980917

[B14] Carloni S. Favrais G. Saliba E. Albertini M. C. Chalon S. Longini M. (2016). Melatonin modulates neonatal brain inflammation through endoplasmic reticulum stress, autophagy, and miR-34a/silent information regulator 1 pathway. *J. Pineal Res.* 61 370–380. 10.1111/jpi.12354 27441728

[B15] Carloni S. Perrone S. Buonocore G. Longini M. Proietti F. Balduini W. (2008). Melatonin protects from the long-term consequences of a neonatal hypoxic-ischemic brain injury in rats. *J. Pineal Res.* 44 157–164. 10.1111/j.1600-079X.2007.00503.x 18289167

[B16] Carloni S. Proietti F. Rocchi M. Longini M. Marseglia L. D’angelo G. (2017). Melatonin pharmacokinetics following oral administration in preterm neonates. *Molecules* 22:E2115. 10.3390/molecules22122115 29194416 PMC6149762

[B17] Carusillo Theriault B. Woo S. K. Karimy J. K. Keledjian K. Stokum J. A. Sarkar A. (2017). Cerebral microbleeds in a neonatal rat model. *PLoS One* 12:e0171163. 10.1371/journal.pone.0171163 28158198 PMC5291518

[B18] Chen Q. Tang J. Tan L. Guo J. Tao Y. Li L. (2015). Intracerebral hematoma contributes to hydrocephalus after intraventricular hemorrhage via aggravating iron accumulation. *Stroke* 46 2902–2908. 10.1161/STROKEAHA.115.009713 26265129

[B19] Chevallier M. Debillon T. Pierrat V. Delorme P. Kayem G. Durox M. (2017). Leading causes of preterm delivery as risk factors for intraventricular hemorrhage in very preterm infants: results of the EPIPAGE 2 cohort study. *Am. J. Obstet. Gynecol.* 216 e511–e518. 10.1016/j.ajog.2017.01.002 28104401

[B20] Cho K. J. Noh S. H. Han S. M. Choi W. I. Kim H. Y. Yu S. (2018). ZMYND10 stabilizes intermediate chain proteins in the cytoplasmic pre-assembly of dynein arms. *PLoS Genet.* 14:e1007316. 10.1371/journal.pgen.1007316 29601588 PMC5895051

[B21] Das A. Wallace G. T. Reiter R. J. Varma A. K. Ray S. K. Banik N. L. (2013). Overexpression of melatonin membrane receptors increases calcium-binding proteins and protects VSC4.1 motoneurons from glutamate toxicity through multiple mechanisms. *J. Pineal Res.* 54 58–68. 10.1111/j.1600-079X.2012.01022.x 22823500 PMC11877314

[B22] Del Bigio M. R. Di Curzio D. L. (2016). Nonsurgical therapy for hydrocephalus: a comprehensive and critical review. *Fluids Barriers CNS* 13:3. 10.1186/s12987-016-0025-2 26846184 PMC4743412

[B23] Delgehyr N. Meunier A. Faucourt M. Bosch Grau M. Strehl L. Janke C. (2015). Ependymal cell differentiation, from monociliated to multiciliated cells. *Methods Cell Biol.* 127 19–35. 10.1016/bs.mcb.2015.01.004 25837384

[B24] Dominguez Rubio A. P. Correa F. Aisemberg J. Dorfman D. Bariani M. V. Rosenstein R. E. (2017). Maternal administration of melatonin exerts short- and long-term neuroprotective effects on the offspring from lipopolysaccharide-treated mice. *J. Pineal Res.* 63:e12439. 10.1111/jpi.12439 28776755

[B25] Edwards J. M. Edwards L. E. Swamy G. K. Grotegut C. A. (2018). Magnesium sulfate for neuroprotection in the setting of chorioamnionitis. *J. Matern. Fetal Neonatal Med.* 31 1156–1160. 10.1080/14767058.2017.1311312 28395549

[B26] Farkas J. Reglodi D. Gaszner B. Szogyi D. Horvath G. Lubics A. (2009). Effects of maternal separation on the neurobehavioral development of newborn Wistar rats. *Brain Res. Bull.* 79 208–214. 10.1016/j.brainresbull.2008.12.011 19150489

[B27] Faubel R. Westendorf C. Bodenschatz E. Eichele G. (2016). Cilia-based flow network in the brain ventricles. *Science* 353 176–178. 10.1126/science.aae0450 27387952

[B28] Fauchere J. C. Koller B. M. Tschopp A. Dame C. Ruegger C. Bucher H. U. (2015). Safety of early high-dose recombinant erythropoietin for neuroprotection in very preterm infants. *J. Pediatr.* 167 52–57.e3. 10.1016/j.jpeds.2015.02.052 25863661

[B29] Feldner A. Adam M. G. Tetzlaff F. Moll I. Komljenovic D. Sahm F. (2017). Loss of Mpdz impairs ependymal cell integrity leading to perinatal-onset hydrocephalus in mice. *EMBO Mol. Med.* 9 890–905. 10.15252/emmm.201606430 28500065 PMC5494508

[B30] Fernandez A. Ordonez R. Reiter R. J. Gonzalez-Gallego J. Mauriz J. L. (2015). Melatonin and endoplasmic reticulum stress: relation to autophagy and apoptosis. *J. Pineal Res.* 59 292–307. 10.1111/jpi.12264 26201382

[B31] Fukumizu M. Takashima S. Becker L. E. (1995). Neonatal posthemorrhagic hydrocephalus: neuropathologic and immunohistochemical studies. *Pediatr. Neurol.* 13 230–234. 8554660 10.1016/0887-8994(95)00183-g

[B32] Fukumizu M. Takashima S. Becker L. E. (1996). Glial reaction in periventricular areas of the brainstem in fetal and neonatal posthemorrhagic hydrocephalus and congenital hydrocephalus. *Brain Dev.* 18 40–45. 8907341 10.1016/0387-7604(95)00103-4

[B33] Gano D. Andersen S. K. Glass H. C. Rogers E. E. Glidden D. V. Barkovich A. J. (2015). Impaired cognitive performance in premature newborns with two or more surgeries prior to term-equivalent age. *Pediatr. Res.* 78 323–329. 10.1038/pr.2015.106 26020148 PMC4540651

[B34] Gao C. Du H. Hua Y. Keep R. F. Strahle J. Xi G. (2014). Role of red blood cell lysis and iron in hydrocephalus after intraventricular hemorrhage. *J. Cereb. Blood Flow Metab.* 34 1070–1075. 10.1038/jcbfm.2014.56 24667910 PMC4050252

[B35] Gluckman P. D. Wyatt J. S. Azzopardi D. Ballard R. Edwards A. D. Ferriero D. M. (2005). Selective head cooling with mild systemic hypothermia after neonatal encephalopathy: multicentre randomised trial. *Lancet* 365 663–670. 15721471 10.1016/S0140-6736(05)17946-X

[B36] Gonzalez F. F. Ferriero D. M. (2008). Therapeutics for neonatal brain injury. *Pharmacol. Ther.* 120 43–53. 10.1016/j.pharmthera.2008.07.003 18718848

[B37] Guirao B. Meunier A. Mortaud S. Aguilar A. Corsi J. M. Strehl L. (2010). Coupling between hydrodynamic forces and planar cell polarity orients mammalian motile cilia. *Nat. Cell Biol.* 12 341–350. 10.1038/ncb2040 20305650

[B38] Hadj Ayed Tka K. Mahfoudh Boussaid A. Zaouali M. A. Kammoun R. Bejaoui M. Ghoul Mazgar S. (2015). Melatonin modulates endoplasmic reticulum stress and Akt/GSK3-beta signaling pathway in a rat model of renal warm ischemia reperfusion. *Anal. Cell. Pathol.* 2015:635172. 10.1155/2015/635172 26229743 PMC4502281

[B39] Hardeland R. (2017). Melatonin and the electron transport chain. *Cell. Mol. Life Sci.* 74 3883–3896. 10.1007/s00018-017-2615-9 28785805 PMC11107625

[B40] Hollund I. M. H. Olsen A. Skranes J. Brubakk A. M. Haberg A. K. Eikenes L. (2018). White matter alterations and their associations with motor function in young adults born preterm with very low birth weight. *Neuroimage Clin.* 17 241–250. 10.1016/j.nicl.2017.10.006 29159041 PMC5683190

[B41] Holwerda J. C. Van Braeckel K. Roze E. Hoving E. W. Maathuis C. G. B. Brouwer O. F. (2016). Functional outcome at school age of neonatal post-hemorrhagic ventricular dilatation. *Early Hum. Dev.* 96 15–20. 10.1016/j.earlhumdev.2016.02.005 26986627

[B42] Hong Z. Hong H. Chen H. Wang Z. Hong D. (2012). Protective effects of erythropoietin in experimental spinal cord injury by reducing the C/EBP-homologous protein expression. *Neurol. Res.* 34 85–90. 10.1179/1743132811Y.0000000026 22196867

[B43] Hu Q. Di G. Shao X. Zhou W. Jiang X. (2018). Predictors associated with post-traumatic hydrocephalus in patients with head injury undergoing unilateral decompressive craniectomy. *Front. Neurol.* 9:337 10.3389/fneur.2018.00337 PMC5960668 29867743

[B44] Hu W. Ma Z. Di S. Jiang S. Li Y. Fan C. (2016). Snapshot: implications for melatonin in endoplasmic reticulum homeostasis. *Br. J. Pharmacol.* 173 3431–3442. 10.1111/bph.13651 27759160 PMC5120159

[B45] Huang Z. Wang Y. Hu G. Zhou J. Mei L. Xiong W. C. (2016). YAP is a critical inducer of SOCS3, preventing reactive astrogliosis. *Cereb. Cortex* 26 2299–2310. 10.1093/cercor/bhv292 26679195 PMC4830299

[B46] Iwai M. Stetler R. A. Xing J. Hu X. Gao Y. Zhang W. (2010). Enhanced oligodendrogenesis and recovery of neurological function by erythropoietin after neonatal hypoxic/ischemic brain injury. *Stroke* 41 1032–1037. 10.1161/STROKEAHA.109.570325 20360553 PMC2919308

[B47] Jantzie L. Oppong A. Conteh F. Yellowhair T. Kim J. Fink G. (2018). Repetitive neonatal erythropoietin and melatonin combinatorial treatment provides sustained repair of functional deficits in a rat model of cerebral palsy. *Front. Neurol.* 9:233. 10.3389/fneur.2018.00233 29706928 PMC5908903

[B48] Jantzie L. L. Corbett C. J. Berglass J. Firl D. J. Flores J. Mannix R. (2014a). Complex pattern of interaction between in utero hypoxia-ischemia and intra-amniotic inflammation disrupts brain development and motor function. *J. Neuroinflammation* 11:131. 10.1186/1742-2094-11-131 25082427 PMC4128546

[B49] Jantzie L. L. Getsy P. M. Firl D. J. Wilson C. G. Miller R. H. Robinson S. (2014b). Erythropoietin attenuates loss of potassium chloride co-transporters following prenatal brain injury. *Mol. Cell. Neurosci.* 61 152–162. 24983520 10.1016/j.mcn.2014.06.009 PMC4134983

[B50] Jantzie L. L. Getsy P. M. Denson J. L. Firl D. J. Maxwell J. R. Rogers D. A. (2015a). Prenatal hypoxia-ischemia induces abnormalities in CA3 microstructure, potassium chloride co-transporter 2 expression and inhibitory tone. *Front. Cell. Neurosci.* 9:347. 10.3389/fncel.2015.00347 26388734 PMC4558523

[B51] Jantzie L. L. Winer J. L. Maxwell J. R. Chan L. A. Robinson S. (2015b). Modeling encephalopathy of prematurity using prenatal hypoxia-ischemia with intra-amniotic lipopolysaccharide in rats. *J. Vis. Exp.* 105:e53196. 10.3791/53196 26649874 PMC4692750

[B52] Jantzie L. L. Miller R. H. Robinson S. (2013). Erythropoietin signaling promotes oligodendrocyte development following prenatal systemic hypoxic-ischemic brain injury. *Pediatr. Res.* 74 658–667. 10.1038/pr.2013.155 24108187 PMC3865073

[B53] Jantzie L. L. Robinson S. (2015). Preclinical models of encephalopathy of prematurity. *Dev. Neurosci.* 37 277–288. 10.1159/000371721 25722056 PMC4514537

[B54] Jantzie L. L. Winer J. L. Corbett C. J. Robinson S. (2016). Erythropoietin modulates cerebral and serum degradation products from excess calpain activation following prenatal hypoxia-ischemia. *Dev. Neurosci.* 38 15–26. 10.1159/000441024 26551007 PMC4732905

[B55] Johnston M. V. Fatemi A. Wilson M. A. Northington F. (2011). Treatment advances in neonatal neuroprotection and neurointensive care. *Lancet Neurol.* 10 372–382. 10.1016/S1474-4422(11)70016-3 21435600 PMC3757153

[B56] Juul S. Mcpherson R. Bauer L. Ledbetter K. Gleason C. Mayock D. (2008). A phase I/II trial of high-dose erythropoietin in extremely low birth weight infants: pharmacokinetics and safety. *Pediatrics* 122 383–391. 10.1542/peds.2007-2711 18676557

[B57] Juul S. E. Comstock B. A. Heagerty P. J. Mayock D. E. Goodman A. M. Hauge S. (2018). High-dose erythropoietin for asphyxia and encephalopathy (HEAL): a randomized controlled trial - background, aims, and study protocol. *Neonatology* 113 331–338. 10.1159/000486820 29514165 PMC5980685

[B58] Karimy J. K. Zhang J. Kurland D. B. Theriault B. C. Duran D. Stokum J. A. (2017). Inflammation-dependent cerebrospinal fluid hypersecretion by the choroid plexus epithelium in posthemorrhagic hydrocephalus. *Nat. Med.* 23 997–1003. 10.1038/nm.4361 28692063

[B59] Kilkenny C. Browne W. J. Cuthill I. C. Emerson M. Altman D. G. (2010). Improving bioscience research reporting: the ARRIVE guidelines for reporting animal research. *PLoS Biol.* 8:e1000412. 10.1371/journal.pbio.1000412 20613859 PMC2893951

[B60] Kim J. Y. Grunke S. D. Levites Y. Golde T. E. Jankowsky J. L. (2014). Intracerebroventricular viral injection of the neonatal mouse brain for persistent and widespread neuronal transduction. *J. Vis. Exp.* 91:51863. 10.3791/51863 25286085 PMC4199253

[B61] Kulkarni A. V. Riva-Cambrin J. Rozzelle C. J. Naftel R. P. Alvey J. S. Reeder R. W. (2018). Endoscopic third ventriculostomy and choroid plexus cauterization in infant hydrocephalus: a prospective study by the Hydrocephalus Clinical Research Network. *J. Neurosurg. Pediatr.* 21 214–223. 10.3171/2017.8.PEDS17217 29243972

[B62] Kwon M. S. Woo S. K. Kurland D. B. Yoon S. H. Palmer A. F. Banerjee U. (2015). Methemoglobin is an endogenous toll-like receptor 4 ligand-relevance to subarachnoid hemorrhage. *Int. J. Mol. Sci.* 16 5028–5046. 10.3390/ijms16035028 25751721 PMC4394463

[B63] Kyrousi C. Arbi M. Pilz G. A. Pefani D. E. Lalioti M. E. Ninkovic J. (2015). Mcidas and GemC1 are key regulators for the generation of multiciliated ependymal cells in the adult neurogenic niche. *Development* 142 3661–3674. 10.1242/dev.126342 26395491

[B64] Lattke M. Magnutzki A. Walther P. Wirth T. Baumann B. (2012). Nuclear factor kappaB activation impairs ependymal ciliogenesis and links neuroinflammation to hydrocephalus formation. *J. Neurosci.* 32 11511–11523. 10.1523/JNEUROSCI.0182-12.2012 22915098 PMC6703776

[B65] Lee J. Romero R. Kim S. M. Chaemsaithong P. Park C. W. Park J. S. (2016). A new anti-microbial combination prolongs the latency period, reduces acute histologic chorioamnionitis as well as funisitis, and improves neonatal outcomes in preterm PROM. *J. Matern. Fetal Neonatal Med.* 29 707–720. 10.3109/14767058.2015.1020293 26373262 PMC5704947

[B66] Leijser L. M. Miller S. P. Van Wezel-Meijler G. Brouwer A. J. Traubici J. Van Haastert I. C. (2018). Posthemorrhagic ventricular dilatation in preterm infants: when best to intervene? *Neurology* 90 e698–e706. 10.1212/WNL.0000000000004984 29367448 PMC5818161

[B67] Leuchter R. H. Gui L. Poncet A. Hagmann C. Lodygensky G. A. Martin E. (2014). Association between early administration of high-dose erythropoietin in preterm infants and brain MRI abnormality at term-equivalent age. *JAMA* 312 817–824. 10.1001/jama.2014.9645 25157725

[B68] Li Z. Li X. Chan M. T. V. Wu W. K. K. Tan D. Shen J. (2017). Melatonin antagonizes interleukin-18-mediated inhibition on neural stem cell proliferation and differentiation. *J. Cell Mol. Med.* 21 2163–2171. 10.1111/jcmm.13140 28429571 PMC5571550

[B69] Lu H. Wang Q. Lu J. Zhang Q. Kumar P. (2016). Risk factors for intraventricular hemorrhage in preterm infants born at 34 weeks of gestation or less following preterm premature rupture of membranes. *J. Stroke Cerebrovasc. Dis.* 25 807–812. 10.1016/j.jstrokecerebrovasdis.2015.12.011 26796051

[B70] Mahuzier A. Shihavuddin A. Fournier C. Lansade P. Faucourt M. Menezes N. (2018). Ependymal cilia beating induces an actin network to protect centrioles against shear stress. *Nat. Commun.* 9:2279. 10.1038/s41467-018-04676-w 29891944 PMC5996024

[B71] Mangano F. T. Altaye M. Mckinstry R. C. Shimony J. S. Powell S. K. Phillips J. M. (2016). Diffusion tensor imaging study of pediatric patients with congenital hydrocephalus: 1-year postsurgical outcomes. *J. Neurosurg. Pediatr.* 18 306–319. 10.3171/2016.2.PEDS15628 27203134 PMC5035704

[B72] Maxwell J. R. Denson J. L. Joste N. E. Robinson S. Jantzie L. L. (2015). Combined in utero hypoxia-ischemia and lipopolysaccharide administration in rats induces chorioamnionitis and a fetal inflammatory response syndrome. *Placenta* 36 1378–1384. 10.1016/j.placenta.2015.10.009 26601766

[B73] Mazur M. Miller R. Robinson S. (2010). Postnatal erythropoietin treatment mitigates neural cell loss after systemic prenatal hypoxic-ischemic injury. *J. Neurosurg. Pediatr.* 6 206–221. 10.3171/2010.5.PEDS1032 20809703 PMC3037962

[B74] Mazzola C. A. Choudhri A. F. Auguste K. I. Limbrick D. D. Jr. Rogido M. Mitchell L. (2014). Pediatric hydrocephalus: systematic literature review and evidence-based guidelines. Part 2: management of posthemorrhagic hydrocephalus in premature infants. *J. Neurosurg. Pediatr.* 14(Suppl. 1), 8–23. 10.3171/2014.7.PEDS14322 25988778

[B75] McAllister J. P. II Williams M. A. Walker M. L. Kestle J. R. Relkin N. R. Anderson A. M. (2015). An update on research priorities in hydrocephalus: overview of the third National Institutes of Health-sponsored symposium “Opportunities for Hydrocephalus Research: pathways to better outcomes”. *J. Neurosurg.* 123 1427–1438. 10.3171/2014.12.JNS132352 26090833

[B76] McAllister J. P. Guerra M. M. Ruiz L. C. Jimenez A. J. Dominguez-Pinos D. Sival D. (2017). Ventricular zone disruption in human neonates with intraventricular hemorrhage. *J. Neuropathol. Exp. Neurol.* 76 358–375. 10.1093/jnen/nlx017 28521038 PMC6251528

[B77] McDougall A. R. A. Hale N. Rees S. Harding R. De Matteo R. Hooper S. B. (2017). Erythropoietin protects against lipopolysaccharide-induced microgliosis and abnormal granule cell development in the ovine fetal cerebellum. *Front. Cell. Neurosci.* 11:224. 10.3389/fncel.2017.00224 28804448 PMC5532439

[B78] Mendivil-Perez M. Soto-Mercado V. Guerra-Librero A. Fernandez-Gil B. I. Florido J. Shen Y. Q. (2017). Melatonin enhances neural stem cell differentiation and engraftment by increasing mitochondrial function. *J. Pineal Res.* 63:e12415. 10.1111/jpi.12415 28423196

[B79] Mirzadeh Z. Han Y. G. Soriano-Navarro M. Garcia-Verdugo J. M. Alvarez-Buylla A. (2010). Cilia organize ependymal planar polarity. *J. Neurosci.* 30 2600–2610. 10.1523/JNEUROSCI.3744-09.2010 20164345 PMC2873868

[B80] Moscuzza F. Belcari F. Nardini V. Bartoli A. Domenici C. Cuttano A. (2011). Correlation between placental histopathology and fetal/neonatal outcome: chorioamnionitis and funisitis are associated to intraventricular haemorrage and retinopathy of prematurity in preterm newborns. *Gynecol. Endocrinol.* 27 319–323. 10.3109/09513590.2010.487619 20528214

[B81] Muir R. T. Wang S. Warf B. C. (2016). Global surgery for pediatric hydrocephalus in the developing world: a review of the history, challenges, and future directions. *Neurosurg. Focus* 41:E11. 27798988 10.3171/2016.7.FOCUS16273

[B82] Mukai T. Mori Y. Shimazu T. Takahashi A. Tsunoda H. Yamaguchi S. (2017). Intravenous injection of umbilical cord-derived mesenchymal stromal cells attenuates reactive gliosis and hypomyelination in a neonatal intraventricular hemorrhage model. *Neuroscience* 355 175–187. 10.1016/j.neuroscience.2017.05.006 28504197

[B83] Muniz-Talavera H. Schmidt J. V. (2017). The mouse Jhy gene regulates ependymal cell differentiation and ciliogenesis. *PLoS One* 12:e0184957. 10.1371/journal.pone.0184957 29211732 PMC5718522

[B84] Ng K. Y. Leong M. K. Liang H. Paxinos G. (2017). Melatonin receptors: distribution in mammalian brain and their respective putative functions. *Brain Struct. Funct.* 222 2921–2939. 10.1007/s00429-017-1439-6 28478550

[B85] Nunez-Olle M. Jung C. Terre B. Balsiger N. A. Plata C. Roset R. (2017). Constitutive Cyclin O deficiency results in penetrant hydrocephalus, impaired growth and infertility. *Oncotarget* 8 99261–99273. 10.18632/oncotarget.21818 29245899 PMC5725090

[B86] Ohls R. K. Cannon D. C. Phillips J. Caprihan A. Patel S. Winter S. (2016). Preschool assessment of preterm infants treated with darbepoetin and erythropoietin. *Pediatrics* 137:e20153859. 10.1542/peds.2015-3859 26908704 PMC4771132

[B87] Ohls R. K. Kamath-Rayne B. D. Christensen R. D. Wiedmeier S. E. Rosenberg A. Fuller J. (2014). Cognitive outcomes of preterm infants randomized to darbepoetin, erythropoietin, or placebo. *Pediatrics* 133 1023–1030. 10.1542/peds.2013-4307 24819566 PMC4531269

[B88] Olsen A. Dennis E. L. Evensen K. A. I. Husby Hollund I. M. Lohaugen G. C. C. Thompson P. M. (2018). Preterm birth leads to hyper-reactive cognitive control processing and poor white matter organization in adulthood. *Neuroimage* 167 419–428. 10.1016/j.neuroimage.2017.11.055 29191480 PMC6625518

[B89] Osier N. D. Pham L. Pugh B. J. Puccio A. Ren D. Conley Y. P. (2017). Brain injury results in lower levels of melatonin receptors subtypes MT1 and MT2. *Neurosci. Lett.* 650 18–24. 10.1016/j.neulet.2017.03.053 28377323 PMC5886725

[B90] Pappas A. Adams-Chapman I. Shankaran S. Mcdonald S. A. Stoll B. J. Laptook A. R. (2018). Neurodevelopmental and behavioral outcomes in extremely premature neonates with ventriculomegaly in the absence of periventricular-intraventricular hemorrhage. *JAMA Pediatr.* 172 32–42. 10.1001/jamapediatrics.2017.3545 29181530 PMC5833521

[B91] Park R. Moon U. Y. Park J. Y. Hughes L. J. Johnson R. L. Cho S. H. (2016). Yap is required for ependymal integrity and is suppressed in LPA-induced hydrocephalus. *Nat. Commun.* 7:10329. 10.1038/ncomms10329 26754915 PMC4729961

[B92] Peng X. Lin Q. Liu Y. Jin Y. Druso J. E. Antonyak M. A. (2013). Inactivation of Cdc42 in embryonic brain results in hydrocephalus with ependymal cell defects in mice. *Protein Cell* 4 231–242. 10.1007/s13238-012-2098-2 23150167 PMC3632363

[B93] Poryo M. Boeckh J. C. Gortner L. Zemlin M. Duppre P. Ebrahimi-Fakhari D. (2017). Ante-, peri- and postnatal factors associated with intraventricular hemorrhage in very premature infants. *Early Hum. Dev.* 116 1–8. 10.1016/j.earlhumdev.2017.08.010 29091782

[B94] Radic J. A. Vincer M. Mcneely P. D. (2015). Outcomes of intraventricular hemorrhage and posthemorrhagic hydrocephalus in a population-based cohort of very preterm infants born to residents of Nova Scotia from 1993 to 2010. *J. Neurosurg. Pediatr.* 15 580–588. 10.3171/2014.11.PEDS14364 26030329

[B95] Ramirez-Jirano L. J. Zenteno-Savin T. Gaxiola-Robles R. Ramos-Gonzalez E. J. Torres-Mendoza B. M. Bitzer-Quintero O. K. (2016). The neuroprotective effect of erythropoietin in rat hippocampus in an endotoxic shock model. *Rev. Invest. Clin.* 68 292–298. 28134940

[B96] Riva-Cambrin J. Kestle J. R. Holubkov R. Butler J. Kulkarni A. V. Drake J. (2016). Risk factors for shunt malfunction in pediatric hydrocephalus: a multicenter prospective cohort study. *J. Neurosurg. Pediatr.* 17 382–390. 10.3171/2015.6.PEDS14670 26636251

[B97] Robertson N. J. Tan S. Groenendaal F. Van Bel F. Juul S. E. Bennet L. (2012). Which neuroprotective agents are ready for bench to bedside translation in the newborn infant? *J. Pediatr.* 160 544–552.4. 22325255 10.1016/j.jpeds.2011.12.052 PMC4048707

[B98] Robinson S. Corbett C. J. Winer J. L. Chan L. A. S. Maxwell J. R. Anstine C. V. (2017). Neonatal erythropoietin mitigates impaired gait, social interaction and diffusion tensor imaging abnormalities in a rat model of prenatal brain injury. *Exp. Neurol.* 302 1–13. 10.1016/j.expneurol.2017.12.010 29288070 PMC5849508

[B99] Robinson S. Winer J. L. Berkner J. Chan L. A. Denson J. L. Maxwell J. R. (2016). Imaging and serum biomarkers reflecting the functional efficacy of extended erythropoietin treatment in rats following infantile traumatic brain injury. *J. Neurosurg. Pediatr.* 17 739–755. 10.3171/2015.10.PEDS15554 26894518 PMC5369240

[B100] Romero L. Ros B. Arraez M. A. Rius F. Gonzalez L. Martin A. (2015). Analysis of risk factors for hydrocephalus development in newborn infants with germinal matrix hemorrhage. *Minerva Pediatr.* 67 401–406. 26377778

[B101] Rumalla K. Letchuman V. Smith K. A. Arnold P. M. (2018). Hydrocephalus in pediatric traumatic brain injury: national incidence, risk factors, and outcomes in 124,444 hospitalized patients. *Pediatr. Neurol.* 80 70–76. 10.1016/j.pediatrneurol.2017.11.015 29429778

[B102] Rypens E. Avni E. F. Dussaussois L. David P. Vermeylen D. Van Bogaert P. (1994). Hyperechoic thickened ependyma: sonographic demonstration and significance in neonates. *Pediatr. Radiol.* 24 550–553. 7724274 10.1007/BF02012729

[B103] Salas A. A. Faye-Petersen O. M. Sims B. Peralta-Carcelen M. Reilly S. D. Mcgwin G. (2013). Histological characteristics of the fetal inflammatory response associated with neurodevelopmental impairment and death in extremely preterm infants. *J. Pediatr.* 163 e651–e652. 10.1016/j.jpeds.2013.03.081 23664630 PMC3744601

[B104] Samantaray S. Sribnick E. A. Das A. Knaryan V. H. Matzelle D. D. Yallapragada A. V. (2008). Melatonin attenuates calpain upregulation, axonal damage and neuronal death in spinal cord injury in rats. *J. Pineal Res.* 44 348–357. 18086148 10.1111/j.1600-079X.2007.00534.x PMC2613550

[B105] Santos M. M. Rubagumya D. K. Dominic I. Brighton A. Colombe S. O’donnell P. (2017). Infant hydrocephalus in sub-Saharan Africa: the reality on the Tanzanian side of the lake. *J. Neurosurg. Pediatr.* 20 423–431. 10.3171/2017.5.PEDS1755 28885096

[B106] Sawamoto K. Wichterle H. Gonzalez-Perez O. Cholfin J. A. Yamada M. Spassky N. (2006). New neurons follow the flow of cerebrospinal fluid in the adult brain. *Science* 311 629–632. 16410488 10.1126/science.1119133

[B107] Semple B. D. Blomgren K. Gimlin K. Ferriero D. M. Noble-Haeusslein L. J. (2013). Brain development in rodents and humans: identifying benchmarks of maturation and vulnerability to injury across species. *Prog. Neurobiol.*106-107 1–16. 10.1016/j.pneurobio.2013.04.001 23583307 PMC3737272

[B108] Shankaran S. Lin A. Maller-Kesselman J. Zhang H. O’shea T. M. Bada H. S. (2014). Maternal race, demography, and health care disparities impact risk for intraventricular hemorrhage in preterm neonates. *J. Pediatr.* 164 1005–1011.e3. 10.1016/j.jpeds.2014.01.036 24589078 PMC4095864

[B109] Simon T. D. Riva-Cambrin J. Srivastava R. Bratton S. L. Dean J. M. Kestle J. R. (2008). Hospital care for children with hydrocephalus in the United States: utilization, charges, comorbidities, and deaths. *J. Neurosurg. Pediatr.* 1 131–137. 10.3171/PED/2008/1/2/131 18352782

[B110] Spassky N. Merkle F. T. Flames N. Tramontin A. D. Garcia-Verdugo J. M. Alvarez-Buylla A. (2005). Adult ependymal cells are postmitotic and are derived from radial glial cells during embryogenesis. *J. Neurosci.* 25 10–18. 15634762 10.1523/JNEUROSCI.1108-04.2005 PMC6725217

[B111] Stark M. J. Hodyl N. A. Belegar V. K. Andersen C. C. (2016). Intrauterine inflammation, cerebral oxygen consumption and susceptibility to early brain injury in very preterm newborns. *Arch. Dis. Child. Fetal Neonatal Ed.* 101 F137–F142. 26265677 10.1136/archdischild-2014-306945

[B112] Strahle J. Garton H. J. Maher C. O. Muraszko K. M. Keep R. F. Xi G. (2012). Mechanisms of hydrocephalus after neonatal and adult intraventricular hemorrhage. *Transl. Stroke Res.* 3 25–38. 10.1007/s12975-012-0182-9 23976902 PMC3750748

[B113] Takagishi M. Sawada M. Ohata S. Asai N. Enomoto A. Takahashi K. (2017). Daple coordinates planar polarized microtubule dynamics in ependymal cells and contributes to hydrocephalus. *Cell Rep.* 20 960–972. 10.1016/j.celrep.2017.06.089 28746879

[B114] Tang A. T. Choi J. P. Kotzin J. J. Yang Y. Hong C. C. Hobson N. (2017). Endothelial TLR4 and the microbiome drive cerebral cavernous malformations. *Nature* 545 305–310. 10.1038/nature22075 28489816 PMC5757866

[B115] Tissir F. Qu Y. Montcouquiol M. Zhou L. Komatsu K. Shi D. (2010). Lack of cadherins Celsr2 and Celsr3 impairs ependymal ciliogenesis, leading to fatal hydrocephalus. *Nat. Neurosci.* 13 700–707. 10.1038/nn.2555 20473291

[B116] Tsai T. H. Lin C. J. Chua S. Chung S. Y. Yang C. H. Tong M. S. (2017). Melatonin attenuated the brain damage and cognitive impairment partially through MT2 melatonin receptor in mice with chronic cerebral hypoperfusion. *Oncotarget* 8 74320–74330. 10.18632/oncotarget.20382 29088788 PMC5650343

[B117] Vidovic D. Harris L. Harvey T. J. Evelyn Heng Y. H. Smith A. G. Osinski J. (2015). Expansion of the lateral ventricles and ependymal deficits underlie the hydrocephalus evident in mice lacking the transcription factor NFIX. *Brain Res.* 1616 71–87. 10.1016/j.brainres.2015.04.057 25960350

[B118] Vollmer B. Lundequist A. Martensson G. Nagy Z. Lagercrantz H. Smedler A. C. (2017). Correlation between white matter microstructure and executive functions suggests early developmental influence on long fibre tracts in preterm born adolescents. *PLoS One* 12:e0178893. 10.1371/journal.pone.0178893 28594884 PMC5464584

[B119] Volpe J. (2009). Brain injury in premature infants: a complex amalgam of destructive and developmental disturbances. *Lancet Neurol.* 8 110–124. 10.1016/S1474-4422(08)70294-1 19081519 PMC2707149

[B120] Wang R. Li J. Duan Y. Tao Z. Zhao H. Luo Y. (2017). Effects of erythropoietin on gliogenesis during cerebral ischemic/reperfusion recovery in adult mice. *Aging Dis.* 8 410–419. 10.14336/AD.2016.1209 28840056 PMC5524804

[B121] Wang Y. C. Wang P. F. Fang H. Chen J. Xiong X. Y. Yang Q. W. (2013). Toll-like receptor 4 antagonist attenuates intracerebral hemorrhage-induced brain injury. *Stroke* 44 2545–2552. 10.1161/STROKEAHA.113.001038 23839500

[B122] Wei S. Luo C. Yu S. Gao J. Liu C. Wei Z. (2017). Erythropoietin ameliorates early brain injury after subarachnoid haemorrhage by modulating microglia polarization via the EPOR/JAK2-STAT3 pathway. *Exp. Cell Res.* 361 342–352. 10.1016/j.yexcr.2017.11.002 29102603

[B123] Wessell A. P. Kole M. J. Cannarsa G. Oliver J. Jindal G. Miller T. (2018). A sustained systemic inflammatory response syndrome is associated with shunt-dependent hydrocephalus after aneurysmal subarachnoid hemorrhage. *J. Neurosurg.* 10.3171/2018.1.JNS172925 [Epub ahead of print]. 29957109

[B124] Wu Y. W. Mathur A. M. Chang T. Mckinstry R. C. Mulkey S. B. Mayock D. E. (2016). High-dose erythropoietin and hypothermia for hypoxic-ischemic encephalopathy: a phase II trial. *Pediatrics* 137:e20160191. 10.1542/peds.2016-0191 27244862

[B125] Xiong G. Elkind J. A. Kundu S. Smith C. J. Antunes M. B. Tamashiro E. (2014). Traumatic brain injury-induced ependymal ciliary loss decreases cerebral spinal fluid flow. *J. Neurotrauma* 31 1396–1404. 10.1089/neu.2013.3110 24749541 PMC4132584

[B126] Xu P. Morrison J. P. Foley J. F. Stumpo D. J. Ward T. Zeldin D. C. (2018). Conditional ablation of the RFX4 isoform 1 transcription factor: allele dosage effects on brain phenotype. *PLoS One* 13:e0190561. 10.1371/journal.pone.0190561 29298325 PMC5752003

[B127] Xue F. Shi C. Chen Q. Hang W. Xia L. Wu Y. (2017). Melatonin mediates protective effects against kainic acid-induced neuronal death through safeguarding ER stress and mitochondrial disturbance. *Front. Mol. Neurosci.* 10:49. 10.3389/fnmol.2017.00049 28293167 PMC5329003

[B128] Yanni D. Korzeniewski S. J. Allred E. N. Fichorova R. N. O’shea T. M. Kuban K. (2017). Both antenatal and postnatal inflammation contribute information about the risk of brain damage in extremely preterm newborns. *Pediatr. Res.* 82 691–696. 10.1038/pr.2017.128 28549057 PMC5599336

[B129] Young C. C. Van Der Harg J. M. Lewis N. J. Brooks K. J. Buchan A. M. Szele F. G. (2013). Ependymal ciliary dysfunction and reactive astrocytosis in a reorganized subventricular zone after stroke. *Cereb. Cortex* 23 647–659. 10.1093/cercor/bhs049 22414771 PMC3563342

[B130] Yuan W. Deren K. E. Mcallister J. P. II Holland S. K. Lindquist D. M. Cancelliere A. (2010). Diffusion tensor imaging correlates with cytopathology in a rat model of neonatal hydrocephalus. *Cerebrospinal Fluid Res.* 7:19. 10.1186/1743-8454-7-19 21054844 PMC2989304

[B131] Yung Y. C. Mutoh T. Lin M. E. Noguchi K. Rivera R. R. Choi J. W. (2011). Lysophosphatidic acid signaling may initiate fetal hydrocephalus. *Sci. Transl. Med.* 3:99ra87. 10.1126/scitranslmed.3002095 21900594 PMC3653407

[B132] Zhang H. Fang X. Huang D. Luo Q. Zheng M. Wang K. (2018). Erythropoietin signaling increases neurogenesis and oligodendrogenesis of endogenous neural stem cells following spinal cord injury both in vivo and in vitro. *Mol. Med. Rep.* 17 264–272. 10.3892/mmr.2017.7873 29115443 PMC5780136

[B133] Zhao C. Li Y. Cao W. Xiang K. Zhang H. Yang J. (2016). Diffusion tensor imaging detects early brain microstructure changes before and after ventriculoperitoneal shunt in children with high intracranial pressure hydrocephalus. *Medicine* 95:e5063. 27759635 10.1097/MD.0000000000005063 PMC5079319

[B134] Zhao H. Wang R. Wu X. Liang J. Qi Z. Liu X. (2015). Erythropoietin delivered via intra-arterial infusion reduces endoplasmic reticulum stress in brain microvessels of rats following cerebral ischemia and reperfusion. *J. Neuroimmune Pharmacol.* 10 153–161. 10.1007/s11481-014-9571-z 25626440

[B135] Zhou Z. W. Li F. Zheng Z. T. Li Y. D. Chen T. H. Gao W. W. (2017). Erythropoietin regulates immune/inflammatory reaction and improves neurological function outcomes in traumatic brain injury. *Brain Behav.* 7:e00827. 10.1002/brb3.827 29201540 PMC5698857

