# Supplementary figures and images for: Extended Combined Neonatal Treatment With Erythropoietin Plus Melatonin Prevents Posthemorrhagic Hydrocephalus of Prematurity in Rats
Source: Front Cell Neurosci. 2018 Sep 25;12:322. doi: 10.3389/fncel.2018.00322 (PMC6167494; doi:10.3389/fncel.2018.00322)

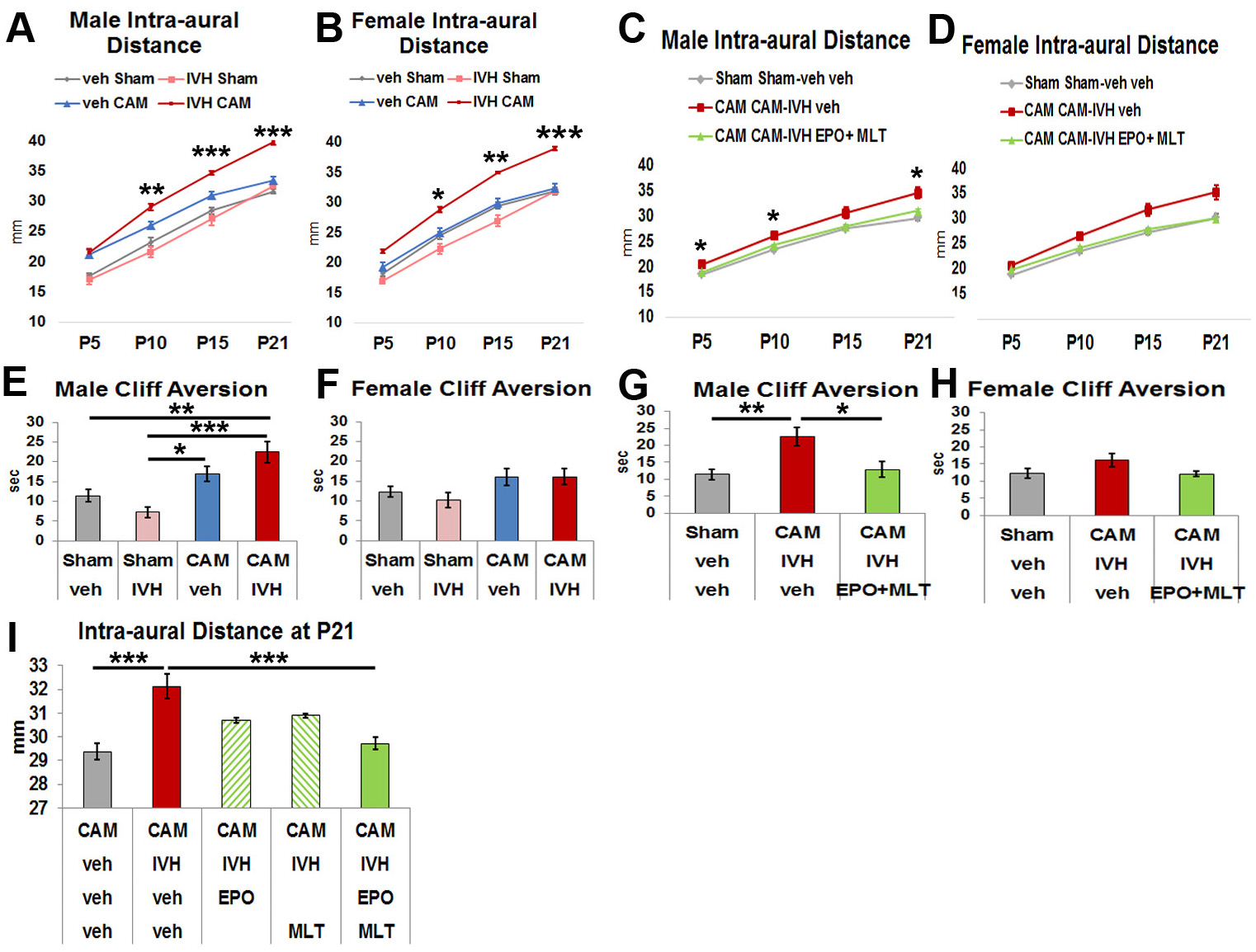

Supplement: FIGURE S1 — Both male and female rats exhibit similar patterns of progressive macrocephaly and poor cliff performance after CAM-IVH, and respond similarly to EPO+MLT treatment. (A) Male CAM-IVH rats (n = 10) show significant macrocephaly from P10 through P21, compared to sham-veh (n = 17), sham-IVH (n = 9), and CAM-veh (n = 10) rats. (B) Similarly, female CAM-IVH rats (n = 12) have larger IADs compared to sham-veh (n = 16), sham-IVH (n = 11), and CAM-veh rats (n = 9). (C) Neonatal EPO+MLT treatment prevents progressive macrocephaly in male CAM-IVH rats (n = 9), compared to vehicle-treated CAM-IVH rats (n = 13). By P21, IADs of male EPO+MLT-treated rats are similar to male shams (n = 15). (D) Similarly, the IADs of female EPO+MLT-treated rats (n = 6) match female shams (n = 13), and diverge from female vehicle-treated CAM-IVH rats (n = 15). (E) For cliff aversion, male CAM-IVH rats (n = 14) perform worse than sham-veh rats (n = 12) and sham-IVH rats (n = 8), while CAM-veh rats (n = 17) also perform poorly. (F) Female CAM-veh (n = 10) and CAM-IVH rats (n = 14) show a similar pattern, compared to sham-veh (n = 12) and sham-IVH (n = 10) rats, but the differences are not statistically significant. (G) Male vehicle-treated CAM-IVH rats (n = 14) perform poorly on cliff aversion compared to sham-veh rats (n = 12) and EPO+MLT–treated CAM-IVH rats (n = 8). (H) While the trend for female vehicle-treated CAM-IVH rats (n = 10) compared to sham-veh (n = 12) and EPO+MLT-treated CAM-IVH rats (n = 9) is similar to the male rats, the differences are not statistically significant. (I) To test the efficacy of EPO alone or MLT alone, CAM-IVH littermates were treated with vehicle (n = 8), EPO alone (n = 9), MLT alone (n = 5), or EPO+MLT (n = 8), and compared to vehicle-treated CAM-veh rats (n = 13). P21 IADs of EPO-treated or MLT-treated CAM-IVH rats were not different than vehicle-treated CAM-IVH rats. By contrast, IADs of EPO+MLT-treated CAM-IVH rats were different than vehicle-treated CAM-IVH rat [file Image_1.JPEG]

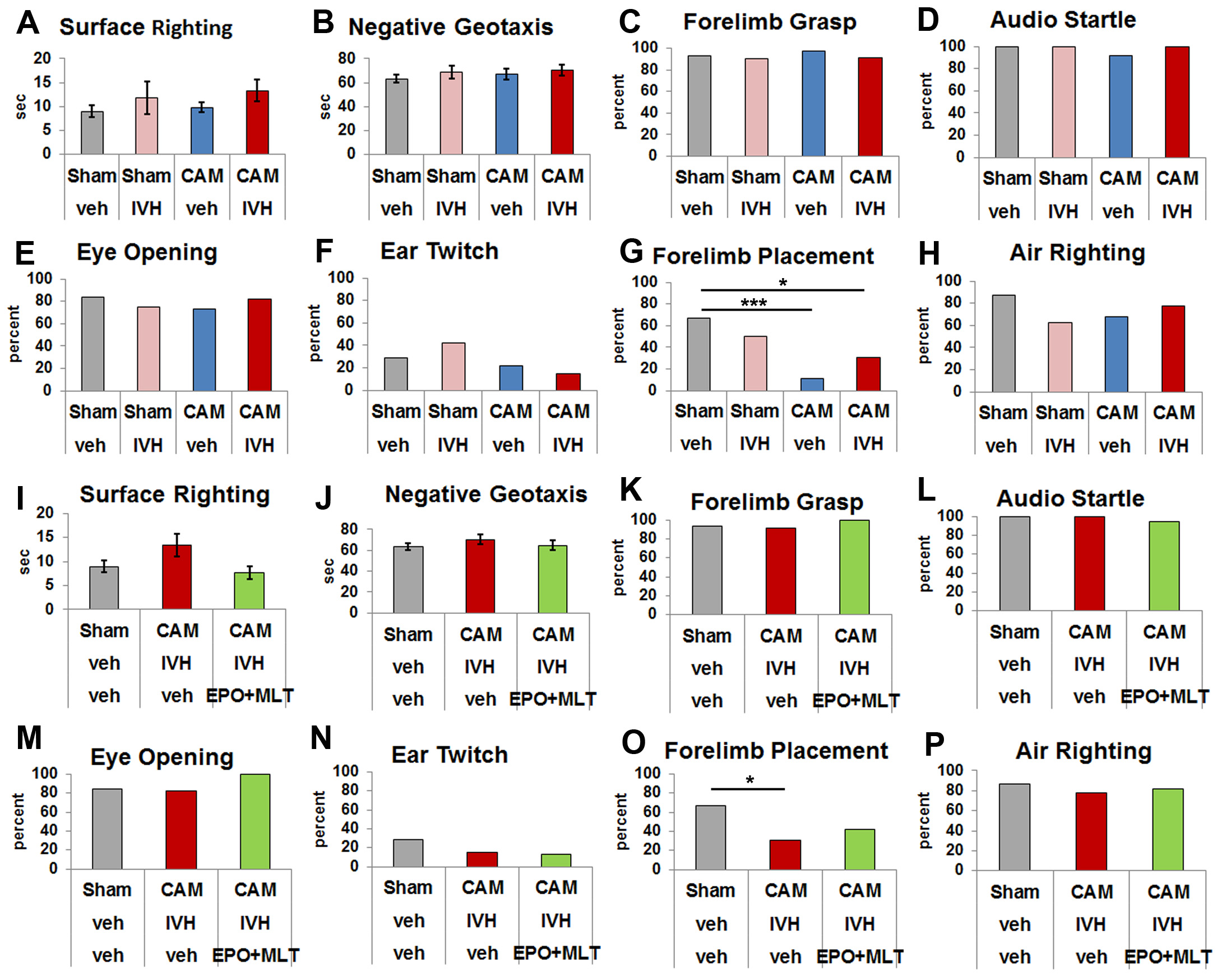

Supplement: FIGURE S2 — Neurobehavioral tests in neonatal rats are minimally affected by CAM-IVH. (A) Surface righting, the sum of the time to turn from lying on the back to upright on feet during three trials from P7 to P9, was mildly impaired in CAM-IVH rats compared to sham-veh rats, but the difference was not significant. The negative geotaxis (B, sum of time to redirect when placed on an inclined plane from pointing head down, with maximum of 30 s), the presence of forelimb grasp on P8 (C), the presence of audio startle on P13 (D), the presence of eye opening on P14 (E), and the presence of ear twitch to cotton wisp on P14 (F) all did not differ between groups. (G) The presence of forelimb placement to stimulus on the dorsum of the forelimb on P15 was reduced in injured rats. (H) The ability to turn upright after being dropped upside down above a cushioned surface on P12 did not differ between groups. (I) Surface righting improved mildly in EPO+MLT-treated CAM-IVH rats compared to vehicle-treated CAM = IVH rats, but the trend was not significant. (J–P) None of the other reflexes differed significantly between the vehicle and EPO+MLT-treated CAM-IVH rats [n = 16–31, two-way ANOVA with Bonferroni correction (A,B,I,J) or Kruskal–Wallis with Dunn’s post hoc correction (C–H,K–P)]. ∗p < 0.05, ∗∗∗p < 0.001. [file Image_2.JPEG]

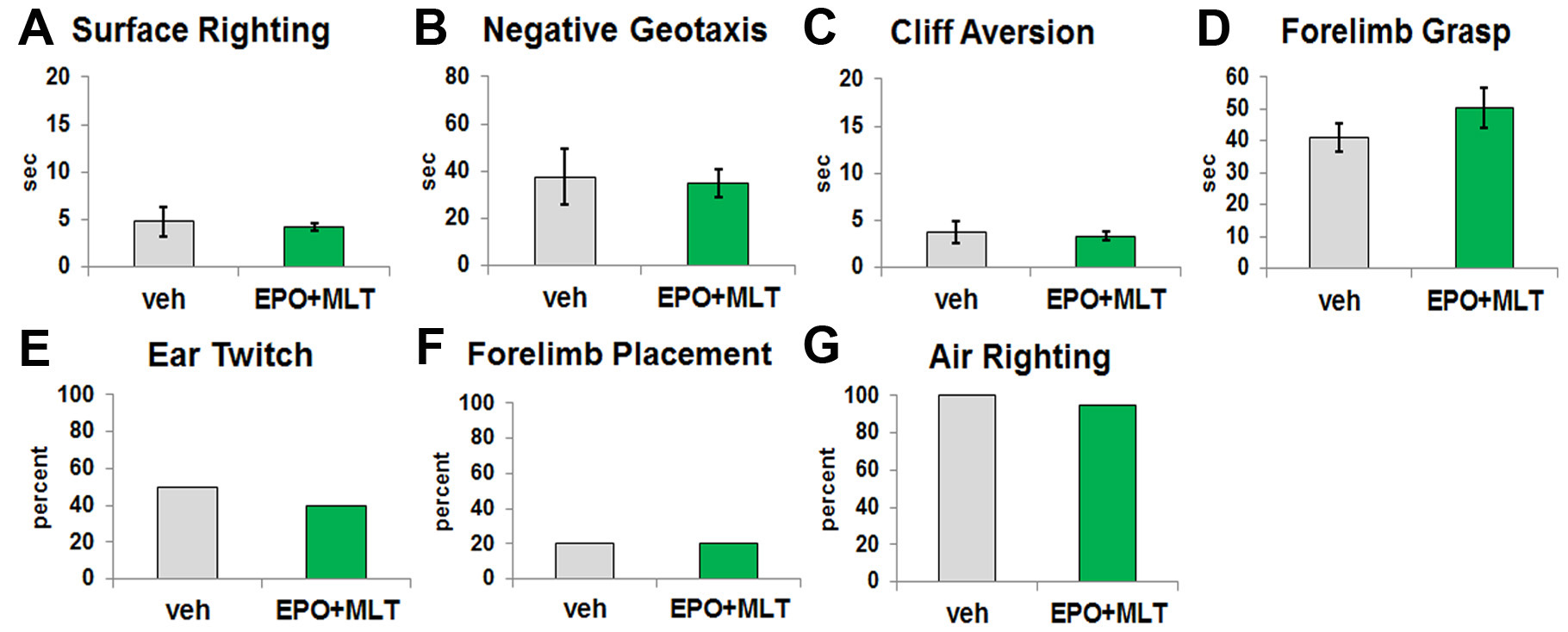

Supplement: FIGURE S3 — Neurodevelopment was assessed in naïve rats treated with EPO+MLT (n = 20) or vehicle (n = 10). Surface righting on P5–P7 (A), negative geotaxis on P8–P14 (B), cliff aversion on P12–P13 (C), forelimb grasp on P10–P11 (D), ear twitch on P14 (E), forelimb placement on P15 (F), and air righting on P12 (G) did not differ between vehicle-treated and EPO+MLT-treated naïve rats. [file Image_3.JPEG]
